# Personality and help-seeking for psychological distress: a systematic review and meta-analysis

**DOI:** 10.3389/fpsyt.2024.1405167

**Published:** 2025-01-23

**Authors:** Anna Szücs, Rachel Hui Xin Lam, Wymann Shao Wen Tang, Lifan Zhou, Monica Lazarus, Andrea B. Maier, Jose M. Valderas

**Affiliations:** ^1^ Department of Medicine, Yong Loo Lin School of Medicine, National University of Singapore, Singapore, Singapore; ^2^ Faculty of Behavioural and Movement Sciences, Vrije Universiteit Amsterdam, Amsterdam, Netherlands; ^3^ Department of Family Medicine, National University Health System, Singapore, Singapore; ^4^ Department of Statistics and Data Science, Faculty of Science, National University of Singapore, Singapore, Singapore; ^5^ Centre for Healthy Longevity, @AgeSingapore, National University Health System, Singapore, Singapore; ^6^ Centre for Research in Health Systems Performance, (CRiHSP) National University of Singapore, Singapore, Singapore

**Keywords:** personality, help-seeking, care seeking, treatment-seeking, social support seeking, depression, anxiety, psychological distress

## Abstract

**Introduction:**

The effective management of depression, anxiety, and other forms of psychological distress depends on individuals’ readiness to seek and accept help for their mental suffering. Understanding which personality traits relate to help-seeking can help better tailor mental healthcare to individual needs. However, findings regarding associations of personality traits with help-seeking have been inconsistent.

**Methods:**

This systematic review and meta-analysis focused on English-language research studies on the association of personality (encompassing personality disorders, Five Factor –Big Five– dimensions, and other measures of personality) with depression, anxiety, or unspecified psychological distress in adults aged 18 years and older. Procedures followed the Preferred Reporting Items for Systematic Reviews and Meta-Analyses (PRISMA) guidelines. The search strategy included two concepts: personality and help-seeking and was carried out on PubMed, Embase, Web of Science, and PsycINFO. Reference tracking and searches on Google Scholar were additionally performed. Sufficiently homogeneous subsections were analyzed by meta-analysis.

**Results:**

A total of 48 studies described in 47 records reported on the association between personality and help-seeking. Nine assessed personality disorders, 29 Five Factor dimensions, and 13 other personality constructs. Twenty-three studies investigated attitudes towards help-seeking while 25 studies investigated help-seeking behaviors. Of the studies investigating behavior, three used external observations, the rest relied on self-reports/clinician-administered questionnaires. Evidence highlighted a dissociation between attitudes and behavior for schizotypal and borderline personality disorders, and neuroticism, which displayed negative help-seeking attitudes but more help-seeking behavior. By contrast, paranoid, schizoid and obsessive-compulsive personality disorders related to both negative help-seeking attitudes and behavior across studies. Limited evidence linked extraversion to social support seeking and conscientiousness to care seeking behaviors. Meta-analyses on the Five Factor dimensions and help-seeking attitudes supported robust negative associations with neuroticism, as well as positive associations with agreeableness, albeit less reliably. Other personality traits mostly corroborated the above relationships, while also contributing new perspectives, such as help-seeking behavior’s negative associations with reality weakness and cynicism, and positive associations with abasement and rigidity.

**Discussion:**

Future research should investigate help-seeking behavior using external observations and longitudinal designs. Assessing personality in clinical settings can help identify populations at risk of keeping to themselves when mentally distressed.

## 1 Introduction

Mental disorders underlying psychological distress such as depression and anxiety are highly prevalent around the world and represent an important burden on populations in terms of role impairment and social costs (1). The effective management of depression, anxiety, and more general forms of psychological distress greatly depends on individuals’ readiness to seek and accept help for their mental suffering in the form of professional care (2) and social support (3). In contrast, failing to seek help has been linked to serious mental illness (4). A tendency to keep one’s psychological distress to oneself has been associated with various social and demographic factors, for instance gender roles (5–7) or racial/ethnic and cultural values (8, 9). However, findings have been inconsistent even regarding well-established sociodemographic determinants of help-seeking for psychological distress, such as gender (10), suggesting that sociodemographic factors do not provide the full picture. Personality has been hypothesized to also play a role in help-seeking (11).

Help-seeking can represent an open admission of dependence on others or of the failure to tackle one’s problems on one’s own, but can also be perceived as the only possible option in cases where one has little faith in one’s own coping abilities (12). Hence, help-seeking may not be perceived as an acceptable solution in case of high individual need for control as in obsessive-compulsive personality disorder and high perfectionism (13), or in case of dominance-driven individual motivations as in narcissistic or antisocial personality profiles (14, 15). By contrast, it could become an overutilized behavioral response to difficulty in individuals who harbor persistent or recurring negative self-views, as in the case of high neuroticism (16) or borderline personality disorder (17). Personality traits may also improve individuals’ help-seeking abilities. For example, extraversion, openness to experience, and agreeableness have been positively associated with communication competence (18), extraversion with seeking social support when facing a challenging task (19), and conscientiousness and openness with clients’ engagement in psychotherapy (20).

Overall, help-seeking has been positively associated with mental health recovery (21), making it a promising strategy to prevent detrimental consequences of psychological distress, such as persisting mental health conditions (22), suicide (23), or long-term disability (24). Yet, randomized controlled interventions promoting help-seeking for psychological distress have shown little effect on objectively measured help-seeking behavior (25), which may be partly due to individual differences such as the above that were not taken into account. Personality-targeted approaches have shown promise in areas where personality risk factors have been consistently identified, such as alcohol use or internalizing and externalizing problems in adolescents (26, 27). Thus, gaining a clearer understanding of the role of personality with respect to help-seeking attitudes and behaviors can represent an essential first step to identify which individuals will spontaneously reach out to healthcare professionals or their social circle in times of difficulty, and which ones will need more proactive, targeted interventions to prevent isolation and downward spirals into more severe psychopathology.

The present systematic review and meta-analysis aims to integrate evidence about the personality disorders and traits associated with help-seeking attitudes and behaviors for psychological distress, defined as depression, anxiety, and unspecific acute psychological stress.

## 2 Methods

We synthesized the research evidence on the association between personality and help-seeking for psychological distress in adult populations. Methods followed the Preferred Reporting Items for Systematic Reviews and Meta-Analyses (PRISMA) guidelines (28). The review’s protocol was preregistered on PROSPERO (29).

### 2.1 Eligibility criteria

#### 2.1.1 Types of records

We included all peer-reviewed quantitative and qualitative studies reporting on original data that were published in English as journal articles or doctoral dissertations. The rationale behind including dissertations was that a majority of studies undertaken in the context of PhD projects in psychology do not get published in journals (30) even though they can be considered peer-reviewed by the thesis committee.

We excluded reviews, expert opinions, case studies, conference abstracts that did not have a full-text version, and records not reporting on original data. We also excluded studies that did not report on the association of interest, namely between personality and help-seeking, or that were not conducted in a human adult population (see below).

#### 2.1.2 Participants

The population of interest was defined as adults of any age, with a cutoff for adulthood at ≥ 18 years of age. Studies including mixed samples of adolescents and adults needed to have their mean age and standard deviation for age ≥ 18 years to be eligible.

#### 2.1.3 Personality: definition and measures

Personality traits were defined according to the American Psychology Association’s Dictionary of Psychology, as “characteristic patterns of thinking, feeling and behaving” (31). Our primary focus was classic measures of personality such as personality disorders as described by the Diagnostic and Statistical Manual of Mental Disorders (DSM) (32) or the International Classification of Diseases (ICD) (33) as well as the five dimensions of personality described in the Five Factor Model (34, 35), namely neuroticism, extraversion, openness to experience, agreeableness, and conscientiousness.

As not all aspects of personality can be captured by the above classifications (36), we additionally included studies investigating other personality constructs as long as they were measured by a psychometric tool that explicitly conceptualized them as trait or personality. This definition relying on the psychometric tool was introduced during full text screening to avoid sampling bias, given a large number of studies in which authors categorized constructs as personality even though they were not systematically defined as such in the literature [for an example: (37)].

#### 2.1.4 Help-seeking for psychological distress: definitions and measures

Help-seeking was defined as either observed or self-reported readiness to seek help for psychological distress from any source, including from professional sources (henceforth referred to as ‘care seeking’) and from social contacts (henceforth referred to as ‘social support seeking’). Help-seeking outcomes encompassed self-reported attitudes towards help-seeking, including help-seeking intentions such as in-principle willingness to seek help in a hypothetical scenario of psychological distress, as well as help-seeking behavior, which could be either self-reported or based on external observations such as medical records or national databases.

As psychological distress is commonly defined as non-specific symptoms of depression, anxiety, and stress (38, 39), we considered cases of depression or anxiety (either defined by self-report or by formal diagnosis) and other, non-specific acute negative emotional states as psychological distress. A similar scope for psychological distress has been employed in another systematic review investigating help-seeking outcomes (25).

Studies reporting on help-seeking, mental healthcare utilization, or consultations for psychosomatic symptoms were eligible as long as they were explicitly investigating these outcomes in the context of psychological distress as defined above. Suicidal ideation and behavior were considered acute negative emotional states, hence eligible. Studies reporting on help-seeking for other mental health conditions were not considered, nor were studies comparing treatment preferences, coping, or problem-solving strategies that did not report specifically on associations between personality and help-seeking for psychological distress. Broadly defined psychological distress such as ‘mental health problems’ remained eligible.

### 2.2 Information sources

The search was conducted using a database combination found to constitute an optimal coverage of the literature (40) that included PubMed, PsycINFO, Web of Science, and Embase with the same set of key words (see next section). We sought additional records through Google Scholar and reference tracking.

### 2.3 Search strategy

#### 2.3.1 Record retrieval and deduplication

The search was first performed in February 2023 and updated in July 2023. Searches were performed in titles and abstracts, using key words grouped under two blocks separated by ‘AND’: personality and help-seeking (**Supplementary Table S1**). The search strategy was piloted on PubMed to refine the search terms, with several terms eliminated due to their lack of contribution to relevant hits. This was the case for the specific names of all personality disorders. The search included no filters or publication date restrictions. Syntax was adapted to each database.

Deduplication took place in EndNote 20 (41), following the procedure described by Bramer and colleagues (42). Records were then imported into Rayyan (43) for title-abstract screening where a second deduplication took place using Rayyan’s built-in tool.

#### 2.3.2 Screening process

Two independent study team members screened each record at both title/abstract and full text screening stages: AS screened all records while RL and WT screened half of the records each. All records selected by at least one screener during title/abstract screening were carried over to full text screening. Discrepancies during full text screening were resolved through consensus meetings with ML acting as tiebreaker.

Each screening phase was preceded by a piloting session where the screening team compared and discussed their decisions on 50 articles for the title/abstract phase and six articles for the full text phase.

Title/abstract screening was carried out using the online software Rayyan whereas full text screening employed EndNote 20 to store all full text articles, with screeners recording full text screening decisions on their individual copy of a standardized Excel spreadsheet.

### 2.4 Data extraction

Two independent study team members extracted data from each record: AS extracted data from all records, while RL and WT extracted data from half of the records each. Discrepant fields were reconciled during meetings between AS, RL, and WT.

### 2.5 Risk of bias assessment

Two independent team members performed a risk of bias assessment (**Supplementary Table S2**) for each record of quantitative studies using the Newcastle-Ottawa Scale (44), and considering the following cutoffs (45): ≥7points, high quality evidence, 4–6 points, moderate quality evidence, and ≤3 points, low quality evidence. An adapted version of the tool for cross-sectional studies used in prior systematic reviews was employed for all cross-sectional studies (46, 47). AS appraised all records. RL and WT appraised half of the records each, after an initial piloting session to clarify the appraisal criteria. Conflicts were resolved during consensus meetings with ML acting as tiebreaker.

### 2.6 Data synthesis methods

Data synthesis followed a narrative summary of all results grouped by personality constructs (DSM/ICD personality disorders, Five Factor Model dimensions, and other personality constructs) and further by outcome (help-seeking attitudes vs behaviors; within behaviors: observed vs self-reported). Studies were organized into tables following the same logic, and ordered following evidence quality within each subcategory.

Suitability for meta-analysis was considered for each of the above sections. Given the scarce number of studies in most subsections, indication for a meta-analysis was only established for studies reporting on associations between Five Factor Model dimensions and help-seeking attitudes, given that most outcome measures in this subsection were adaptations of the same scale, the Attitudes Towards Seeking Professional Psychological Help Scale (48).

We employed random-effects meta-analyses using correlation coefficients between each Five Factor Model dimension and scores on the help-seeking attitudes scales. In studies where only multivariate results were reported, we estimated correlation coefficients based on adjusted beta coefficients in order to factor out variations in effect size due to the use of difference sets of covariates (49). As this method could be subject to biased estimates (50), we conducted an additional sensitivity analysis including only studies that reported correlation coefficients (**Supplementary Figure S1**). In case of significant heterogeneity in the main meta-analysis models, we planned on performing subgroup analyses using the following classifications: sample size, population, country, gender, and race.

## 3 Results

### 3.1 Overview and study characteristics

#### 3.1.1 Screening overview

**Figure 1:**
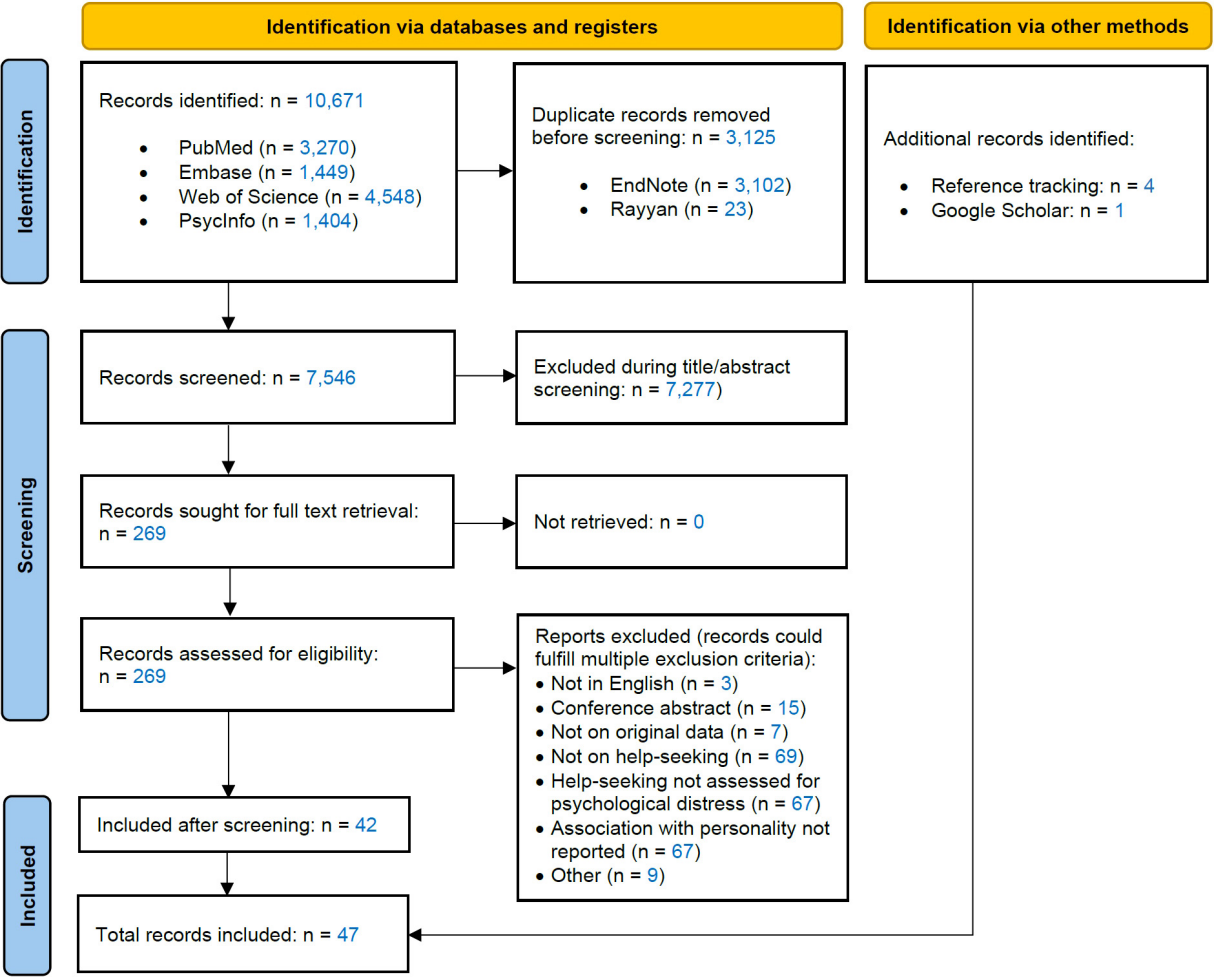
Preferred Reporting Items for Systematic Reviews and Meta-Analyses (PRISMA) flow diagram.

**Figure 1** summarizes the screening process. The initial search yielded 10,671 records, of which 7,546 remained after deduplication. Of these, title/abstract screening selected 269 for full text screening. Several records met more than one criteria for exclusion during full text screening (**Figure 1**). Full text screening resulted in the inclusion of 42 records. Reference tracking provided four additional ones, while the search on Google Scholar provided one additional record.

#### 3.1.2 Final set of records

In total, 47 records were included (51–97), of which nine were doctoral dissertations (53, 57, 58, 71, 76, 81–83, 96). Publication dates ranged from 1967 to 2022, with 10 records published in or after 2020, 32 records between 2010 and 2019, six records between 2000 and 2009, six records between 1990 and 1999, and three records before that.

Twenty-two records were from North America, namely the United States (n = 20) and Canada (n = 2); 15 records were from Europe, including the Netherlands (n = 4), Germany (n = 4), Ireland (n = 2), Norway (n = 2), the United Kingdom (n = 2), and Sweden (n = 1); five records were from the Middle East, including Turkey (n = 2), India (n = 1), Israel (n = 1), and Tunisia (n = 1); three records were from Oceania (n = 3; all from Australia); the remaining two records were from West Africa (n = 1; Nigeria) and East Asia (n = 1; South Korea), respectively.

One record reported on two eligible studies (74), hence the total number of studies included in the review was 48. For clarity, we refer to the number of studies instead of the number of records from this point on.

#### 3.1.3 Study characteristics

Two studies had a qualitative design (87, 88), the rest were quantitative. Of the 46 quantitative studies, one followed a prospective design (90) and 45 were cross-sectional.

The samples used in most studies were college/university students (n = 18), followed by community-dwelling participants (n = 12), other specific populations (based on profession, ethnicity, or other sociodemographic factors; n = 11), and psychiatric populations (n = 8). Two records reported on mixed samples of students and community-dwelling participants (61, 84). Notably, five out of the nine doctoral dissertations reported on student samples (57, 71, 76, 81, 82).

#### 3.1.4 Assessment of personality

Overall, nine studies assessed personality as DSM/ICD personality disorders (Subsection 3.3.1.; **Tables 1**–**3**), 29 as Five Factor dimensions (Subsection 3.3.2.; **Tables 4**, **5**), and 13 as other personality constructs (Subsection 3.3.3.; **Tables 6**, **7**). Two studies (68, 84) assessed Eysenck’s three personality dimensions (98), neuroticism, extroversion, and psychoticism. The two first dimensions were grouped with the Five Factor Model’s neuroticism and extraversion respectively, given these constructs’ considerable overlap (99), whereas psychoticism was reported with other personality traits.

**Table 1:** Studies testing associations between DSM/ICD personality disorders grouped under Cluster A and outcomes measuring help-seeking attitudes and behaviors.

Help-seeking	First author, year, reference	Country	Population	Sample size	Mean age, years (SD)	Sex - % Female	Personality assessment tool	Outcome measure (tool, if applicable)	Association with outcome			RoB
Cluster A: odd or eccentric personality disorders									paranoid	schizoid	schizo-typal	
attitude	Fekih-Romdhane 2021 (64)	Tunisia	college students with high/low schizotypy scores	101	21.1 (1.7)	65.7%	SPQ	ATSPPH-SF			-	***
	Eurelings-Bontekoe 1997 (63)	Netherlands	psychiatric patients	230	36.8 (11.3)	67.8%	VKP	seeking social support as coping in hypothetical scenario (UCL scale item)	- ^a^	- ^a^	- ^a^	**
behavior	Iza 2013 (70)	USA	community-dwelling adults	34,653	NR	NR	AUDADIS-IV	lifetime care seeking for anxiety disorders	- ^bc^	- ^bc^	Ø	***
	Blanch 2021 (54)	Australia	college students	800	21.0 (NR)	76.8%	SPQ	help-seeking for anxiety symptoms ≤ last 8 weeks			+	**

All studies in the table had a cross-sectional design. Studies are organized by outcome type (attitude vs behavior) and risk of bias (from high to low evidence quality). In the ‘Association with outcome’ column, green cells indicate positive associations, pink cells negative associations, grey cells non-significant associations, and black cells that the association was not tested. RoB, Risk of bias assessment with the Newcastle-Ottawa scale for cross-sectional studies; ***, high quality evidence (low risk of bias); **, moderate quality evidence (moderate risk of bias); NR, not reported; ^a-c^, association tested while controlling for ^a^, age and sex/gender; ^b^, race; ^c^, age of onset of psychopathology; SPQ, Schizotypal personality questionnaire; VKP, Vragenlijst voor Kenmerken van de Persoonlijkheid (Questionnaire on Personality Traits); AUDADIS-IV, Alcoholism’s Alcohol Use Disorder and Associated Disabilities Interview Schedule–DSM-IV Version; UCL, Utrecht Coping List; ATSPPH-SF, Attitudes Towards Seeking Professional Psychological Help Scale – Short Form.

**Table 2:** Studies testing associations between DSM/ICD personality disorders grouped under Cluster B and outcomes measuring help-seeking attitudes and behaviors.

Help-seeking	First author, year, reference	Country	Population	Sample size	Mean age, years (SD)	Sex - % Female	Personality assessment tool	Outcome measure (tool, if applicable)	Association with outcome				RoB
Cluster B: emotional or erratic personality disorders									antisocial/dissocial	borderline	histrionic	narcissistic	
attitude	Eurelings-Bontekoe 1997 (63)	Netherlands	psychiatric patients	230	36.8 (11.3)	67.8%	VKP	seeking social support as coping in hypothetical scenario (UCL scale item)	- ^a^	- ^a^	Ø	Ø	**
behavior	Iza 2013 (70)	USA	community-dwelling adults	34,653	NR	NR	AUDADIS-IV	lifetime care seeking for anxiety disorders	- ^bc^	+ ^bc^	Ø	Ø	***
	Broadbear 2020 (56)	Australia	suicide decedents	2870	NR	24.8%	medical records	care seeking ≤ 6 weeks prior to death by suicide ^#^		+			***
	Yasmeen 2022 (94)	USA	jail inmates	506	32,4 (10.0)	30%	PAI-BOR	requests for jail-based treatment ^#^		+ ^ab^			***
	Ullrich 2009 (91)	United Kingdom	community-dwelling adolescents & adults	1051	56.3 (15.2)	68%	SCID-II	care seeking ≤ 12 months	+				***
	Tomko 2014 (89)	USA	community-dwelling adults	34,481	NR	NR	AUDADIS-IV	lifetime care seeking for low mood		+			**
both	Svanborg 2008 (88)	Sweden	patients with non-remitting depression	10	NR	60%	DIP-Q	care seeking (qualitative interview)	*qualitative findings: see main text*				NA

All studies in the table had a cross-sectional design. Studies are organized by outcome type (attitude vs behavior vs both) and risk of bias (from high to low evidence quality). In the ‘Association with outcome’ column, green cells indicate positive associations, pink cells negative associations, grey cells non-significant associations, and black cells that the association was not tested. RoB, Risk of bias assessment with the Newcastle-Ottawa scale for cross-sectional studies; ***, high quality evidence (low risk of bias); **, moderate quality evidence (moderate risk of bias); NA, not applicable; NR, not reported; ^#^, outcome data based on external observations (not self-reports); ^a-c^, association tested while controlling for ^a^, age and sex/gender; ^b^, race; ^c^, age of onset of psychopathology; VKP, Vragenlijst voor Kenmerken van de Persoonlijkheid (Questionnaire on Personality Traits); AUDADIS-IV, Alcoholism’s Alcohol Use Disorder and Associated Disabilities Interview Schedule–DSM-IV Version; PAI-BOR, Personality Assessment Inventory-Borderline Scale; SCID-II, Structured Clinical Interview for DSM-IV Axis II Personality Disorders; DIP-Q, DSM-IV and ICD-10 Personality Questionnaire; UCL, Utrecht Coping List.

**Table 3:** Studies testing associations between DSM/ICD personality disorders grouped under Cluster C or pertaining to former DSM/ICD classifications and outcomes measuring help-seeking attitudes and behaviors.

Help-seeking	First author, year, reference	Country	Population	Sample size	Mean age, years (SD)	Sex - % Female	Personality assessment tool	Outcome measure (tool, if applicable)	Association with outcome							RoB
Cluster C: anxious or fearful personality disorders									avoidant		dependent			obsessive-compulsive		
attitude	Eurelings-Bontekoe 1997 (63)	Netherlands	psychiatric patients	230	36.8 (11.3)	68%	VKP	seeking social support as coping in hypothetical scenario (UCL scale item)	Ø		Ø			- ^a^		**
behavior	Iza 2013 (70)	USA	community-dwelling adults	34,653	NR	NR	AUDADIS-IV	lifetime care seeking for anxiety disorders	- ^bc^		Ø			- ^bc^		***
both	Svanborg 2008 (88)	Sweden	patients with non-remitting depression	10	NR	60%	DIP-Q	care seeking (qualitative interview)	*qualitative findings: see main text*							NA
Additional personality disorders from earlier classifications (DSM-III, ICD-10)									passive-aggressive	self-defeating		sadistic	anxious		impulsive	
attitude	Eurelings-Bontekoe 1997 (63)	Netherlands	psychiatric patients	230	36.8 (11.3)	68%	VKP	seeking social support as coping in hypothetical scenario (UCL scale item)	Ø	- ^a^		Ø	Ø		Ø	**

All studies in the table had a cross-sectional design. Studies are organized by outcome type (attitude vs behavior vs both) and risk of bias (from high to low evidence quality). In the ‘Association with outcome’ column, pink cells indicate negative associations, grey cells non-significant associations, and black cells that the association was not tested. RoB, Risk of bias assessment with the Newcastle-Ottawa scale for cross-sectional studies; ***, high quality evidence (low risk of bias); **, moderate quality evidence (moderate risk of bias); NA, not applicable; NR, not reported; ^a-c^, association tested while controlling for ^a^, age and sex/gender; ^b^, race; ^c^, age of onset of psychopathology; VKP, Vragenlijst voor Kenmerken van de Persoonlijkheid (Questionnaire on Personality Traits); AUDADIS-IV, Alcoholism’s Alcohol Use Disorder and Associated Disabilities Interview Schedule–DSM-IV Version; DIP-Q, DSM-IV and ICD-10 Personality Questionnaire; UCL, Utrecht Coping List.

**Table 4:** Studies testing associations between five factor personality dimensions and outcomes measuring help-seeking attitudes.

First author, year, reference	Country	Population	Sample size	Mean age, years (SD)	Sex - % Female	Tool measuring personality	Outcome measure	Association with outcome					RoB
								N	E	O	A	C	
Hatchett, 2019 (66)	USA	college students	458	20.3 (3.6)	80%	NEO Five Factor Inventory	ATSPPH	Ø	+ ^acf^	+ ^acf^	+ ^acf^	+ ^acf^	***
Puma, 1996 (T) (82)	USA	college students	263	18.6 (1.2)	54%	NEO Five Factor Inventory	ATSPPH	Ø	Ø	Ø	Ø	Ø	***
Kakhnovets, 2011 (72)	USA	college students	411	18.8 (2.4)	47%	NEO Personality Inventory - Revised	ATSPPH-SF	+ M	+ F	+	Ø	Ø	***
Miller, 2010 (T) (76)	USA	college students	784	19.6 (2.1)	61%	IPIP Five Factor Model	ATSPPH-SF	+ ^e^	Ø				***
Oluyinka, 2011 (79)	Nigeria	college students	452	23.3 (2.7)	48%	Openness to Experience scale	ATSPPH-SF			+ ^e^			***
Drapeau, 2016 (62)	worldwide; 82.3% USA	adults bereaved by suicide	418	49.5 (13.1)	90%	NEO Five Factor Inventory	IASMHS	- ^c^	- ^c^	+ ^c^	+ ^c^	Ø	***
Joyce, 2013 (T) (71)	USA	college students	494	NR	60%	IPIP Five Factor Model	ISCI	Ø	Ø	+ ^c^	+ ^c^	Ø	***
Billingsley, 1999 (T) (53)	USA	non-professionals & professionals at a rehabilitation hospital	132	47.9 (4.0)	63%	NEO Five Factor Inventory	ATSPPH	Ø	Ø	+ ^af^	Ø	Ø	**
Yelpaze, 2020 (95)	Turkey	college students	1,284	NR	58%	Basic Personality Traits Inventory	ATSPPH	Ø	Ø	- ^ce^	+ ^ce^	+ ^ce^	**
Ingram, 2016 (69)	USA	primary care patients	227	43.1 (12.8)	41%	Mini IPIP	ATSPPH-SF	Ø	+	Ø	+	Ø	**
Rankine, 2021 (T) (83)	USA	community-dwelling adults	200	34.2 (9.2)	41%	Mini IPIP	ATSPPH-SF	Ø	Ø				**
Samuel, 2022 (85)	Canada	college students	167	NR	78%	Big Five Inventory	IASMHS	Ø	Ø	Ø	Ø	Ø	**
Hyland, 2015 (68)	Ireland	active and retired policemen	331	28.4 (8.6)	39%	Eysenck Personality Questionnaire Revised	IASMHS	+	Ø				**
Kessler, 2015 (73)	Germany	community-dwelling older adults	156	71.5 (6.4)	57%	NEO Five Factor Inventory-30	IASMHS	- ^abfeg^		- ^abfeg^			**
Atik, 2011 (52)	Turkey	college students	524	20.0 (2.1)	76%	Big Five Inventory	SASPH-S	Ø	+ ^af^	+ ^af^	+ ^af^	+	**
O’Connor, 2014 (78)	Australia	college students	180	NR	82%	Big Five Inventory	ISPHQ		+ ^efg^				**

All studies in the table had a cross-sectional design. Studies are organized by risk of bias (from low to high) and outcome measure. In the ‘Association with outcome’ column, green cells indicate positive associations, pink cells negative associations, grey cells non-significant associations, and black cells that the association was not tested. N, neuroticism; E, extraversion; O, openness to experience; A, agreeableness; C, Conscientiousness; RoB, Risk of Bias assessment with the Newcastle-Ottawa scale adapted for cross-sectional studies; ***, high quality evidence (low risk of bias); **, moderate quality evidence (moderate risk of bias); (T), PhD thesis dissertation; NR, not reported; ^a-g^, association tested while controlling for ^a^, gender/sex, ^b^, other demographics, ^c^, the Five Factors and/or other personality traits, ^d^, psychiatric diagnosis/mood, ^e^, other psychological constructs, ^f^, mental health knowledge and/or experience, ^g^, social support; IPIP, International Personality Item Pool; ATSPPH, Attitudes Towards Seeking Professional Psychological Help Scale; ATSPPH-SF, Attitudes Towards Seeking Professional Psychological Help Scale – Short Form; IASMHS, Attitudes Toward Seeking Mental Health Services; ISCI, Intentions to Seek Counseling Inventory; ISPHQ, Intention to Seek Professional Help Questionnaire; M, in men only; F, in women only.

**Table 5:** Studies testing associations between five factor personality dimensions and outcomes measuring help-seeking behavior.

First author, year, reference	Country	Population	Sample size	Mean age, years (SD)	Sex - % Female	Tool measuring personality	Outcome measure	Association with outcome					RoB
								N	E	O	A	C	
Delay in care seeking													
Gormley, 1998 (65)	Ireland	psychiatric patients	82	41.1 (NR)	57%	Maudsley Personality Inventory	time to initial medical consult for depression ^#^	+					***
Valipay, 2019 (92)	India	psychiatric patients with depression	100	NR	54%	Big Five Inventory-10	early vs late help-seeking for depression	Ø	Ø	Ø	Ø	Ø	**
Presence vs absence of care seeking													
Cuijpers, 2007 (59)	Netherlands	nursing home residents	350	84.7 (6.2)	72%	NEO Five Factor Inventory	care seeking for depression ≤ 3 months (ATCWD item)	+ ^abcde^	Ø	Ø	Ø	+ ^d^	***
Schomerus, 2013 (86)	Germany	adults with depression	354	NR	NR	NEO Five Factor Inventory-30	lifetime care seeking for depressive symptoms	Ø	Ø	Ø	Ø	+ ^abcd ef^	***
Boerema, 2016 (55)	Netherlands	adults with depression	102	52 (NR)	54%	NEO Five Factor Inventory	care seeking for major depression ≤ 6 months	Ø					***
van Zoonen, 2015 (93)	Netherlands	adults with subclinical depression	162	57.2 (17.8)	56%	NEO-Five Factor Inventory	care seeking for psychological problems ≤ 6 months	+					***
Park, 2017 (80)	South Korea	community-dwelling adults	1544	NR	54%	Big Five Inventory-10	lifetime care seeking for mental health difficulties	+ ^abd^	Ø	+ ^abd^	- ^abd^	Ø	***
Maier, 1992 (97)	Germany	community-dwelling adults with depression	447	41. (NR)	54%	Munich Personality Test	lifetime and one-year care seeking for depression	Ø	- ^d^				**
Hayslip, 2010 (67)	USA	community-dwelling older adults	233	73.1 (7.7)	66%	NEO Personality Inventory-S	lifetime care seeking for emotional/mental problems	+ R	Ø	+ U			*
Shahaf-Oren, 2021 (87)	United Kingdom	medical students with health issues	11	23.1 (NR)	46%	qualitative interviews	seeking mental health care as medical students	*qualitative findings: see main text*					NA
Social support seeking													
McCrae, 1986 (74)	USA	community-dwelling adults	151	NR	47%	NEO Inventory	seeking social support as coping ≤ 1 year… from checklist (Study 1) freely reported (Study 2) (Ways of Coping Checklist – modified by authors)	Ø	+ ^g^ S2	Ø			**
Rim, 1986 (84)	Israel	college students & community-dwelling adults	174	NR	46%	Eysenck Personality Questionnaire	seeking social support as coping (Ways of Coping Checklist Revised)	+ M	+ F				*

All studies in the table had a cross-sectional design. In the ‘Association with outcome’ column, green cells indicate positive associations, pink cells negative associations, grey cells non-significant associations, and black cells that the association was not tested. N, neuroticism; E, extraversion; O, openness to experience; A, agreeableness; C, Conscientiousness; RoB, Risk of Bias assessment with the Newcastle-Ottawa scale adapted for cross-sectional studies; ***, high quality evidence (low risk of bias); **, moderate quality evidence (moderate risk of bias); *, low quality of evidence (high risk of bias) (T), PhD thesis dissertation; NA, not applicable; NR, not reported; ^#^, outcome data based on external observations (not self-reports); ^a-g^, association tested while controlling for ^a^, gender/sex, ^b^, other demographics, ^c^, chronic illness/comorbidities, ^d^, psychiatric diagnosis/mood, ^e^, other psychological constructs, ^f^, social support, ^g^, mental health knowledge and/or experience; R, only in rural subsample; U, only in urban subsample; M, in men only; F, in women only; S2, in Study 2 only; ATCWD, Actions To Cope With Depression questionnaire.

**Table 6:** Studies testing associations of other personality traits with outcomes measuring help-seeking attitudes.

First author, year	Country	Population	Sample size	Mean age, years (SD)	Sex - % Female	Personality trait: definition (measuring tool)	Outcome measure	Association with outcome	RoB
Cortese, 2004 (58) (T)	USA	male university employees	308	44.3 (12.2)	0%	maladaptive psychological traits (Personality Adjective Check List)	ATSPPH	Ø ^a^	***
Yi, 1998 (96) (T)	USA	Korean Americans	157	31.9 (9.9)	55%	psychological maladjustment: *social alienation, emotional disturbance* (OPI-PI)	ATSPPH	Ø	**
Yelpaze, 2020 (95)	Turkey	college students	1,284	NR	58%	negative valence: *negative self-view* (Basic Personality Traits Inventory)	ATSPPH	- ^ab^	**
Dang, 2020 (61)	Canada	college students & community-dwelling adults	376	23.3 (4.6)	61%	perfectionism traits: *tendency to seek perfection in all tasks/actions* (Multidimensional Perfectionism Scale)	ATSPPH	- S	**
Cole, 2014 (57) (T)	USA	male college students	366	20.2 (2.8)	0%	trait hope: *tendency to be hopeful* (Trait Hope Scale – Revised)	ATSPPH	+ M	**
Pugh, 2002 (81) (T)	USA	college students	281	18.4 (1.9)	50%	interpersonal affect: *emotional, tender* (Jackson Personality Inventory)	ATSPPH-SF	+	**
						tolerance: *broadminded, impartial* (Jackson Personality Inventory)		+	
						other traits of the same inventory (Jackson Personality Inventory)		Ø	
Hyland, 2015 (68)	Ireland	active and retired policemen	331	28.4 (8.6)	39%	psychoticism: *risk-taking, impulsivity, antisocial behavior, non-conformity* (Eysenck Personality Questionnaire Revised)	IASMHS	Ø	**

Studies in the table had a cross-sectional design except for Tyssen, 2004, which was prospective. In the ‘Association with outcome’ column, green cells indicate positive associations, pink cells negative associations, grey cells non-significant associations, and black cells that the association was not tested. Legend: RoB, Risk of Bias assessment with the Newcastle-Ottawa scale adapted for cross-sectional studies; ***, high quality evidence (low risk of bias); **, moderate quality evidence (moderate risk of bias); (T), PhD thesis dissertation; NR, not reported; ^a-b^, association tested while controlling for ^a^, other psychological constructs, ^b^, other personality traits; M, in men only; S, in student subsample; OPI-PI, Omnibus Personality Inventory – Personal Integration subscale; ATSPPH, Attitudes Towards Seeking Professional Psychological Help Scale; ATSPPH-SF, Attitudes Towards Seeking Professional Psychological Help Scale – Short Form; IASMHS, Attitudes Toward Seeking Mental Health Services.

**Table 7:** Studies testing associations of other personality traits with past help-seeking behavior.

First author, year	Country	Population	Sample size	Mean age, years (SD)	Sex - % Female	Personality trait: *definition* (measuring tool)	Outcome measure	Association with outcome	RoB
Dalum, 2022 (60)	Norway	veterinarians	3,464	NR	70%	reality weakness personality trait: *proneness to thoughts/perceptions in-between reality and fantasy* (Torgersen’s Basic Character Inventory)	care seeking for mental health problems ≤ 1 year	-	***
Tyssen, 2004 (90)	Norway	physicians during last year of medical school and at their first and fourth year post medical school	631	28.0 (2.8)	57%	reality weakness personality trait: *proneness to thoughts/perceptions in-between reality and fantasy* (Torgersen’s Basic Character Inventory)	care seeking for mental health problems ≤ 1 year	- ^abcd^	***
						vulnerability: *similar to neuroticism* intensity: *similar to extraversion* control: *compulsiveness, orderliness* (Torgersen’s Basic Character Inventory)		Ø	
Arbisi, 2022 (51)	USA	national guard soldiers	40	31.2 (8.7)	NR	cynicism: *tendency to view others as motivated by self interest* (MMPI2-RC3)	one-year post-deployment mental health service utilization	- ^def^	***
Michal, 2011 (75)	Germany	community-dwelling adolescents & adults	2,495	48.7 (17.4)	55%	Type D Personality: *tendency towards negative affectivity and social inhibition* (Type D Scale-14)	help-seeking and care seeking from various sources ≤ 1 year	+	***
Maier, 1992 (97)	Germany	community-dwelling adults with depression	447	41.1 (NR)	54%	rigidity: *obsessionality* (Munich Personality Test)	lifetime and one-year care seeking for depression	+ ^d^	**
						schizoidia: *isolation & esoteric tendencies* (Munich Personality Test)		Ø	
Minge, 1967 (77)	USA	college students	125	NR	NR	abasement: *tendency to accept blame for problems and confess errors* (Edwards Personal Preference schedule)	counseling clients vs non-clients	+	**
						dominance: *need to lead/influence others* (Edwards Personal Preference schedule)		-	
						order: *need to plan and be organized* (Edwards Personal Preference schedule)		-	
						other traits of the same inventory (Edwards Personal Preference schedule)		Ø	
Yi, 1998 (96) (T)	USA	Korean Americans	157	31.9 (9.9)	55%	psychological maladjustment: *social alienation, emotional disturbance* (OPI-PI)	Help Seeking Behavior Scale	Ø	**
Rim, 1986 (84)	Israel	students & community-dwelling adults	174	NR	46%	psychoticism: *risk-taking, impulsivity, antisocial behavior, non-conformity* (Eysenck Personality Questionnaire)	seeking social support as coping	- F	*

Studies in the table had a cross-sectional design except for Tyssen, 2004, which was prospective. In the ‘Association with outcome’ column, green cells indicate positive associations, pink cells negative associations, grey cells non-significant associations, and black cells that the association was not tested. RoB, Risk of Bias assessment with the Newcastle-Ottawa scale adapted for cross-sectional studies; ***, high quality evidence (low risk of bias); **, moderate quality evidence (moderate risk of bias); (T), PhD thesis dissertation; NR, not reported; ^a-f^, association tested while controlling for ^a^, gender/sex, ^b^, other demographics, ^c^, social support, ^d^, psychiatric diagnosis/mood, ^e^, other psychological constructs, ^f^, mental health knowledge and/or experience; F, in women only; MMPI2-RC3, Minnesota Multiphasic Personality Inventory 2 – Restructured Clinical Scale 3; OPI-PI, Omnibus Personality Inventory – Personal Integration subscale.

Of the nine studies on DSM/ICD personality disorders, one study used medical records to assess personality (56), the others used fully structured, clinician-administered questionnaires (70, 89, 91), or self-reports (54, 63, 64, 88, 94). Assessments of the Five Factor Model dimensions and of other personality traits employed self-reports.

#### 3.1.5 Assessment of help-seeking

About the same number of studies investigated attitudes towards help-seeking (n = 23) and past help-seeking behaviors (n = 25); one quantitative study (96) and one qualitative study (88) investigated both types of outcomes.

One study among those assessing help-seeking attitudes (63), and four studies among those assessing past help-seeking behaviors (54, 74, 75, 84) investigated social support seeking. Most other studies (n = 43) investigated care seeking, with one study reporting on both types of help-seeking (75). Information was lacking about the type of help-seeking in one study (92).

All 23 studies reporting on help-seeking attitudes used self-reports. The most commonly used self-report scales were the 29-item Attitudes Towards Seeking Professional Psychological Help (ATSPPH) questionnaire (48) used in eight studies (53, 57, 58, 61, 66, 82, 95, 96), its 10-item short form (100) used in seven studies (64, 69, 72, 76, 79, 81, 83), and the 24-item Inventory of Attitudes towards Seeking Mental Health Services (101), a modified version of the ATSPPH, used in four studies (62, 68, 73, 85).

The majority of the 25 studies investigating help-seeking behavior relied on self-reports, with only three studies measuring help-seeking through external observations such as official records (94), or collateral history from relatives and/or mental healthcare providers (56, 65). One study measured help-seeking behavior as part of a structured interview and used external data sources as confirmation where available (97). One study left unclear whether it assessed care seeking by self-reports or counseling center records (77). With respect to the past help-seeking behavior’s timeframe, 15 studies set a limited timeframe, which ranged from six weeks (56) to one year (51, 60, 74, 75, 90, 91, 97), or was determined by context, e.g., years spent in medical school (87) or in jail (94). Six studies investigated lifetime help-seeking behavior (67, 70, 80, 86, 89, 97), whereas the timeframe was not specified in one study (84) and one study assessed both one year and lifetime help-seeking (97). Two studies assessed delays in care seeking (65, 92).

### 3.2 Assessment of risk of bias

About half of the quantitative studies (23 out of 46) were rated as high evidence quality (low risk of bias). Most other studies were rated as moderate evidence quality (n = 21), whereas two studies were rated as low evidence quality (67, 84).

Appraisal categories where a majority of studies showed concern included outcome reliability (n = 42), as the outcome was measured as self-report in most studies; comparability of findings, as many studies did not adjust their analysis to mental health status or other potential confounders (n = 32), and reporting on non-respondents (n = 28). Detailed scores for the risk of bias assessment can be found in **Supplementary Table S2**. Risk of bias ratings have also been added to **Tables 1**–**7** for ease of reference.

### 3.3 Synthesized findings by personality construct

#### 3.3.1 DSM/ICD personality disorders

**Tables 1**–**3** provide a summary of studies reporting on the association between DSM/ICD Personality Disorders and help-seeking attitudes (n = 2), help-seeking behavior (n = 6), or both (n = 1).

##### 3.3.1.1 Cluster A personality disorders

As indicated in **Table 1**, four studies reported on the association between Cluster A personality disorders, namely paranoid, schizoid, and schizotypal personality disorders, and help-seeking (54, 63, 64, 70), with two of them investigating schizotypal personality alone (54, 64).

All three personality disorders were associated with more negative attitudes towards social support seeking (63) and schizotypal traits were also associated with more negative attitudes towards seeking professional psychological help (64).

However, whereas paranoid and schizoid personality disorders were also associated with a lower likelihood of having sought care for anxiety disorders in a community sample, this association was not found with schizotypal personality disorder (70), which was linked to an increased likelihood of recent (≤ 8 weeks) help-seeking for anxiety symptoms in college students (54).

##### 3.3.1.2 Cluster B personality disorders

As indicated in **Table 2**, one qualitative (88) and six quantitative studies (56, 63, 70, 89, 91, 94) reported on associations between help-seeking and Cluster B personality disorders, namely antisocial [labeled ‘dissocial’ in ICD-10 (102)], borderline, histrionic, and narcissistic personality disorders: two studies investigated all four Cluster B traits (63, 70), whereas other studies reported associations with antisocial (91) or borderline traits (56, 88, 89, 94).

In the only study investigating attitudes towards help-seeking for psychological distress, antisocial and borderline personality disorders were associated with more negative attitudes (63). Patients with non-remitting depression and borderline traits suggested that the tendency to conceal their feelings contributed to their negative attitudes and delayed help-seeking (88).

However, all associations between borderline personality disorder/traits and past help-seeking behaviors were positive, indicating that individuals with more borderline traits were more likely to seek professional help for anxiety or low mood during their life (70, 89), but also in more acutely stressful situations, such as a suicidal crisis (56) or during time spent in jail (94).

Findings were more inconsistent for antisocial personality disorder, with studies reporting a lower likelihood of lifetime care seeking for anxiety disorders (70) but a higher likelihood of one-year care seeking for more generally defined psychological distress (91).

Limited evidence found no associations between histrionic and narcissistic personality disorders and attitudes towards help-seeking (63) or past help-seeking behavior (70).

##### 3.3.1.3 Cluster C personality disorders

As indicated in **Table 3** (upper), one qualitative (88) and two quantitative studies (63, 70) reported on associations between help-seeking and Cluster C personality disorders, namely avoidant, dependent, and obsessive-compulsive personality disorders.

Of the three personality disorders, only obsessive-compulsive had a negative association with attitudes towards help-seeking (63).

Both avoidant and obsessive-compulsive personality disorders had negative associations with lifetime care seeking for anxiety disorders (70). In a qualitative study, patients with non-remitting depression and avoidant personality traits further indicated that their difficulties with handling conflict could hinder their engagement in therapy (88).

##### 3.3.1.4 Personality disorders from former classifications (DSM-III/ICD-10)

As also indicated in **Table 3** (lower), one study investigated former personality disorder diagnoses, namely passive-aggressive, self-defeating, sadistic, anxious, and impulsive personality disorders (63). It found that self-defeating personality was associated with more negative attitudes towards help-seeking, yet no associations were found for the other personality disorders.

#### 3.3.2 Five factor personality dimensions

A total of 16 studies investigated associations between Five Factor dimensions and help-seeking attitudes (**Table 4**), all of which investigated attitudes towards care seeking (52, 53, 62, 66, 68, 69, 71–73, 76, 78, 79, 82, 83, 85, 95), whereas 13 studies contained in 12 records tested associations with help-seeking behaviors [**Table 5** (55, 59, 65, 67, 74, 80, 84, 86, 87, 92, 93)]. Of these, ten studies focused on care seeking and three studies from two records investigated social support seeking (74, 84).

##### 3.3.2.1 Neuroticism

With respect to help-seeking attitudes, three studies out of the 14 investigating neuroticism found significant positive associations (68, 72, 76), with one of them only finding this association in men (72). Two other studies, which adjusted their analysis to confounders such as the four other Five Factor dimensions or sociodemographic characteristics and mental health experience found negative associations (62, 73). Of note, the negative associations were found in studies with older study samples (mean age ≥ 49.5 years) compared to studies reporting positive associations (mean age ≤ 28.6 years).

Neuroticism was positively associated with help-seeking behavior in six out of eleven studies. Care seeking was associated with neuroticism in community-dwelling adults (80), rural older adults (67), nursing home residents (59), and subclinically depressed adults (93), whereas social support seeking was positively associated with neuroticism in a male subsample of college students and community-dwelling adults (84). However, one study out of two also linked neuroticism to a longer delay before seeking care in psychiatric patients (65).

##### 3.3.2.2 Extraversion

Of the 14 studies investigating associations between extraversion and help-seeking attitudes, five found positive associations, namely in primary care patients (69), college students (52, 66, 78), and female college students only (72). One study on adults bereaved following suicide found a negative association between extraversion and help-seeking attitudes (62).

One out of five studies found a negative association between extraversion and care seeking (97), whereas two out of three studies found positive associations between extraversion and social support seeking (74, 84), with one study only finding this association in women (84).

##### 3.3.2.3 Openness to experience

Seven out of twelve studies investigating the relationship between help-seeking attitudes and openness to experience found positive associations (52, 53, 62, 66, 71, 72, 79), whereas two found negative associations (73, 95). Positive as well as negative associations were reported in both student samples and older populations. However, studies reporting positive associations had overall higher evidence quality.

Openness to experience had a positive association with lifetime care seeking for mental health difficulties in community dwelling adults (80) and in urban (but not rural) older adults (67). In a qualitative study, medical students with physical and/or mental health issues also named openness as an important personality trait to be able to disclose mental suffering to healthcare professionals (87). There were no associations reported with other types of help-seeking behaviors.

##### 3.3.2.4 Agreeableness

Of the ten studies investigating agreeableness and attitudes towards help-seeking, positive associations were found in six studies with diverse populations, namely college students (52, 66, 71, 95), adults bereaved by suicide (62), and primary care patients (69), whereas none reported negative associations.

With respect to help-seeking behaviors, a negative association between agreeableness and lifetime care seeking for mental health difficulties was found in a Korean population study with a large sample (N = 1,544) and high evidence quality (80), but in none of the other three studies investigating this relationship (59, 86, 92).

##### 3.3.2.5 Conscientiousness

Three out of ten studies found a positive relationship between conscientiousness and attitudes towards help-seeking, all of which were conducted in college students (52, 66, 95) and two of which were carried out in Turkey (52, 95). No study found negative associations.

With respect to help-seeking behaviors, two studies found positive associations with care seeking for depression, within three months in nursing home residents (59) and at any point in life in depressed adults (86).

##### 3.3.2.6 Confirmation of main trends by meta-analysis for help-seeking attitudes

**Figure 2:**
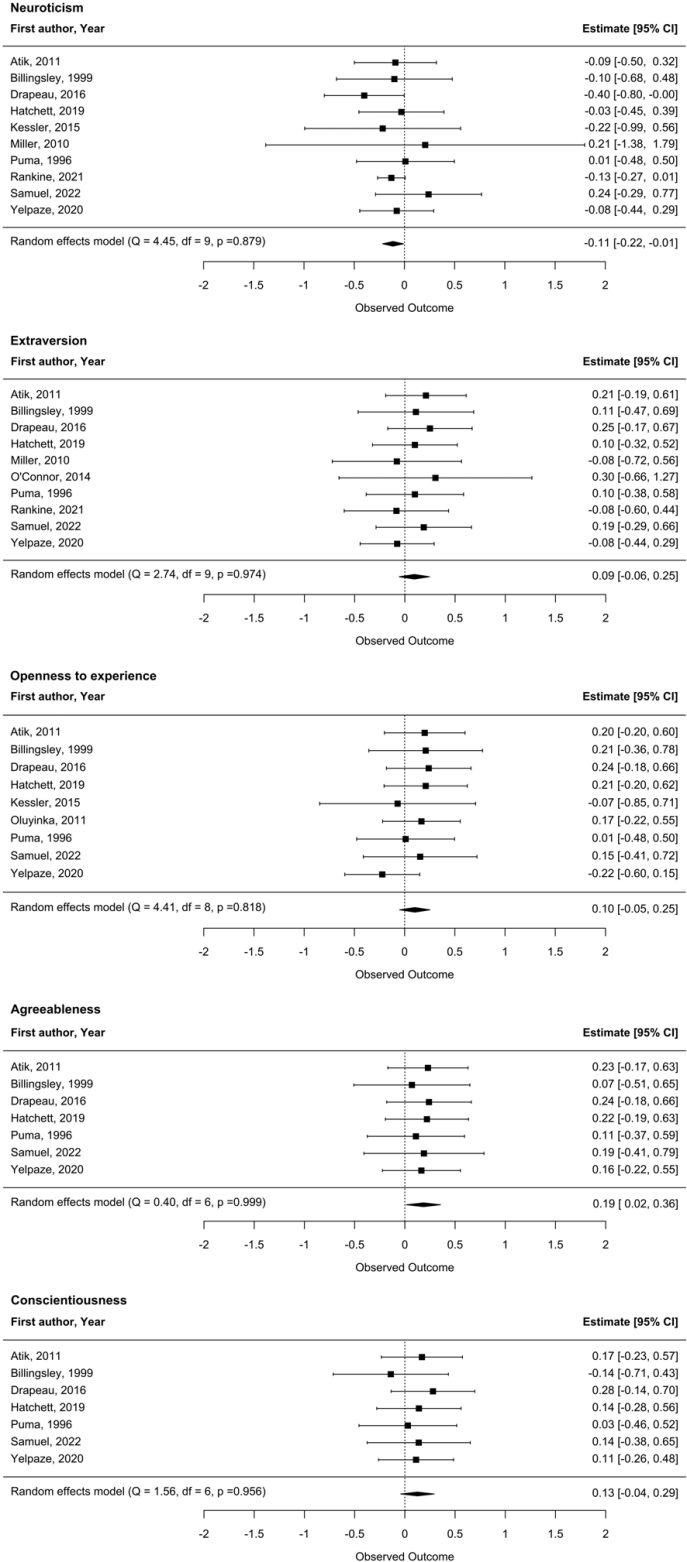
Meta-analysis results (Forest plots) of associations between the Five Factor dimensions of personality and attitudes towards professional help seeking. Estimates indicate correlation coefficients (reported by studies or estimated based on standardized beta coefficients).

Random-effects meta-analyses for the Five Factor dimensions (**Figure 2**) did not show significant heterogeneity, hence we did not conduct any subgroup analysis. The main analysis supported the presence of modest associations between neuroticism and more negative attitudes towards seeking professional psychological help as well as between agreeableness and more positive attitudes towards seeking professional psychological help.

Of these two associations, only the one with neuroticism remained in a sensitivity analysis excluding studies for which correlation coefficients were estimated based on beta coefficients (**Supplementary Figure S1**).

#### 3.3.3 Other personality traits not belonging to DSM/ICD personality disorders or to the Five Factor Model

Of the 14 studies assessing personality constructs not included in DSM/ICD personality disorders or in the Five Factor Model, seven investigated attitudes towards help-seeking [**Table 6** (57, 58, 61, 68, 81, 95, 96)] and eight investigated help-seeking behavior [**Table 7** (51, 60, 75, 77, 84, 90, 96, 97)]. One study investigated both types of outcomes (96).

With respect to attitudes towards help-seeking, negative associations were present in college students who had higher levels of negative valence (negative self-view) (95) and more perfectionistic traits (61). By contrast, traits related to a positive mindset such as trait hope, interpersonal affect (the tendency to be emotional and tender, close to the Five Factor Model’s agreeableness), and tolerance (the tendency to be broadminded and impartial, close to the Five Factor Model’s openness to experience) had positive associations with help-seeking attitudes (57, 81), although the association with trait hope was only present in men (57).

With respect to help-seeking behavior, care seeking for mental health difficulties was negatively associated with reality weakness (the tendency to experience thoughts/perceptions in-between reality and fantasy, especially when feeling overwhelmed by situations) in Norwegian samples of veterinarians (60) and early-career physicians over a four-year follow up period (90). By contrast, abasement, the tendency to accept blame and confess errors was positively associated with care seeking in college students (77).

Traits related to interpersonal functioning, namely cynicism, the tendency to view others as motivated by their own interest (51), and dominance, the need to lead/influence others (77) were also related to less care seeking, whereas psychoticism, defined by risk-taking, impulsivity, and non-conformity, was related to less social support seeking (84).

Order, which is seen by some authors as a dimension of perfectionism (103) and conscientiousness (104) and is defined by the need to be organized and to plan ahead, was also negatively associated with care seeking in college students (77). However, rigidity, a trait characterizing obsessionality and linked to order, perfectionism (105), and obsessive-compulsive personality disorder (106, 107), was positively associated with lifetime and one-year treatment-seeking for depression (97).

Finally, Type D personality, which encompasses negative affectivity and social inhibition, was positively associated with both help- and care seeking in a community sample (75).

## 4 Discussion

### 4.1 Summary of main findings

This work reviewed the evidence on associations between personality and help-seeking for psychological distress. Of the 48 studies reporting on this association, about half investigated help-seeking behaviors as opposed to attitudes towards help-seeking. Most studies used self-reports to assess help-seeking and less than half provided high quality evidence.

With respect to DSM/ICD personality disorders, limited cross-sectional evidence indicated opposite associations of attitudes and behaviors with schizotypal and borderline personality disorders, which were related to increased help-seeking behavior despite more negative attitudes towards help-seeking. In contrast, avoidant, obsessive-compulsive, paranoid, and schizoid personality disorders were associated with less help-seeking behavior, and with more negative attitudes towards help-seeking in the case of the latter three. Surprisingly, neither histrionic nor narcissistic personality disorders were associated with help-seeking attitudes or behaviors, despite their established associations with emotional liability and with increased general health care utilization in the case of histrionic personality disorder (108, 109). However, as only two studies reported on each disorder, this lack of associations may primarily reflect a scarcity of research, which should be addressed.

Of the Five Factor personality dimensions, neuroticism was the only one that was associated with negative attitudes towards professional care seeking. This association was confirmed by meta-analysis and is generally consistent with findings from other studies linking neuroticism to poorer disease self-management (110), and to the use of more maladaptive coping strategies in response to depression and anxiety (111). At the same time, neuroticism was associated with more help-seeking behavior (care seeking in particular) across most studies, suggesting a similar dissociation between attitudes and behavior as observed for some DSM/ICD personality disorders. The four ‘adaptive’ Five Factor dimensions manifested trends of positive attitudes towards professional care seeking, although they only reached significance for agreeableness in the meta-analysis, and did not hold after removing estimated correlation coefficients. Extraversion was positively associated with social support seeking, but had a negative association with care seeking. By contrast, conscientiousness was positively associated with care seeking and had no association with social support seeking.

Associations reported for other personality constructs corroborated several above-mentioned relationships with help-seeking attitudes. Negative attitudes towards help-seeking were linked to negative valence, which is close to the self-consciousness facet of neuroticism (104), and to perfectionism, a dimension of obsessive-compulsive personality disorder (32). Associations with help-seeking behavior supported findings of DSM/ICD personality disorders and the Five Factor Model, e.g., the negative relationship between help-seeking behavior and cynicism, which is included in paranoid personality (32, 33), or the positive associations with Type D personality, closely related to neuroticism (112).

Yet, findings with other personality constructs and help-seeking behavior sometimes nuanced results obtained with DSM/ICD personality disorders or the Five Factor Model. Reality weakness, commonly associated with paranoid, schizotypal, and borderline personality disorders (113, 114), had negative associations with help-seeking behavior, suggesting that escaping into fantasy when feeling overwhelmed may contribute to an avoidance of seeking help in the two Cluster A disorders, but not necessarily in borderline personality disorder, where other symptoms may play a larger part in getting into mental health care.

Similarly, rigidity’s positive association with help-seeking behavior suggests that the negative relationship between help-seeking behavior and obsessive-compulsive personality disorder may arise from different underlying dimensions. Even though rigidity has been defined as a form of perfectionism (105), these two constructs have been identified as distinct factors of obsessive-compulsive personality disorder (106, 107), with perfectionism corresponding to orderliness, and rigidity mapping more clearly on stubbornness (107). As rigidity has been associated with depression above and beyond other personality traits such as neuroticism (105), it may be the case that it reflects a particularly maladaptive side of obsessive-compulsive personality disorder, which is independent, or only partially overlapping with perfectionism.

### 4.2 Integration of findings regarding help-seeking attitudes and behavior

The integrated evidence highlights a dissociation between certain individuals’ reservations towards seeking help for psychological distress and their actual help-seeking behavior. This appears to be the case for adults with high neuroticism, a strong predictor of prospective risk of depression and anxiety (115), as well as schizotypal and borderline personality disorders, identified as the most robust independent predictors of persisting major depression among all DSM personality disorders (116). It seems likely that these personality traits/disorders tie in with dysfunctional behavioral patterns, which reduce emotional expressivity on the one hand (117–119), while increasing emotional instability and subsequent mental healthcare use on the other.

By contrast, some of the personality constructs that displayed a consistent pattern of negative attitudes towards help-seeking and less past help-seeking behavior may delineate groups that remain undertreated for acute psychological distress. Based on scarce evidence, such patterns may be present for paranoid and schizoid personality disorders, and possibly for obsessive-compulsive personality disorder, although a contrasting positive relationship between rigidity and help-seeking behavior weakens this last supposition.

Consistent with the notion of under-treatment, paranoid and schizoid personality disorders are scarce in mental healthcare settings. Adding to the challenge, their prognosis remains poorer than for other personality disorders when admitted for inpatient treatment (120). Obsessive-compulsive personality disorder has been linked to death by suicide in old age (121), which most often arises in the context of depression (122), and may signal failure at seeking help (123).

Associations with personality traits facilitating the help-seeking process remained inconsistent, possibly due to a lack of adjustment for mental illness presence and/or severity. Moreover, it remains likely that personality traits may compensate for each other (124), and studying their combinations would yield more consistent results.

Finally, it appears necessary to test personality in relation to objective measures of help-seeking, namely externally observed, longitudinal help-seeking outcomes. Only one third of individuals who report positive attitudes towards help-seeking for serious emotional problems will seek professional psychological help over the following 10 years (125). Moreover, intervention studies promoting help-seeking for psychological distress tend to impact help-seeking attitudes but fail to obtain results on help-seeking behavior (25). Looking at help-seeking through the lens of personality can help understand broader behavioral patterns underlying help-seeking behavior and personalize motivational approaches to a greater extent. Personality measures have already been integrated into machine learning algorithms for risk/outcome prediction (126), suggesting their relevance for precision mental health.

### 4.3 Strengths and limitations

Strengths of the present systematic review and meta-analysis include its robust methodology and comprehensive synthesis of a broad range of personality constructs and outcomes that enabled a more nuanced understanding of the dynamics between personality and attitudinal vs behavioral assessments of help-seeking for psychological distress.

With respect to limitations, it is worth noting that the definitions of personality and psychological distress were limited to a relatively narrow scope. Further, as only three studies assessing help-seeking behavior consistently relied on external observations and only one used longitudinal data, most reported relationships retain non-negligible subjectivity, even though observed and prospective outcomes aligned with most self-reported, cross-sectional associations. Prior research has found considerable recall bias for self-reported ambulatory physician visits over the past year (127). The heterogeneity of findings in most subsections did not make it possible to conduct meta-analyses. Finally, the limited number of studies with similar characteristics made it challenging to draw conclusions about differences between sampled populations on age, sex, culture, psychopathology, and other factors.

## 5 Conclusions

Many pathological and maladaptive personality disorders and traits have been linked to negative attitudes towards seeking psychological help, in particular from professionals. Personality profiles characterized by high neuroticism, schizotypal, and borderline traits may be more likely to engage in mental healthcare despite negative general attitudes towards care seeking, whereas others, such as schizoid, paranoid, or obsessive-compulsive personality disorders will more likely remain concealed from potential sources of help despite unfavorable long-term prognostics. Future research should confirm prominent findings with more longitudinal evidence and objectively measured outcomes, while clinical interventions should consider focusing efforts on hard-to-reach populations, such as individuals with paranoid, schizoid, or obsessive-compulsive traits. Traits that can be leveraged in interventions aimed at improving help-seeking may include extraversion for social support seeking, conscientiousness for care seeking, and agreeableness for more positive attitudes towards help-seeking.

## References

[1] Kessler RC Aguilar-Gaxiola S Alonso J Chatterji S Lee S Ormel J. The global burden of mental disorders: An update from the WHO World Mental Health (WMH) Surveys. Epidemiol Psichiatr Soc. (2009) 18:23–33. doi: 10.1017/S1121189X00001421 19378696 PMC3039289

[2] Ghio L Gotelli S Cervetti A Respino M Natta W Marcenaro M. Duration of untreated depression influences clinical outcomes and disability. J Affect Disord. (2015) 175:224–8. doi: 10.1016/j.jad.2015.01.014 25658495

[3] Buckman JEJ Saunders R O’Driscoll C Cohen ZD Stott J Ambler G. Is social support pre-treatment associated with prognosis for adults with depression in primary car. Acta Psychiatr Scand. (2021) 143:392–405. doi: 10.1111/acps.v143.5 33548056 PMC7610633

[4] Kessler RC Berglund PA Bruce ML Koch JR Laska EM Leaf PJ. The prevalence and correlates of untreated serious mental illness. Health Serv Res. (2001) 36:987–1007. 11775672 PMC1089274

[5] House J Marasli P Lister M Brown JSL. Male views on help-seeking for depression: A Q methodology study. Psychol Psychother Theory Res Pract. (2018) 91:117–40. doi: 10.1111/papt.2018.91.issue-1 29087607

[6] Judd F Komiti A Jackson H. How does being female assist help-seeking for mental health problems? Aust N Z J Psychiatry. (2008) 42:24–9. doi: 10.1080/00048670701732681 18058440

[7] Staiger T Stiawa M Mueller-Stierlin AS Kilian R Beschoner P Gündel H. Masculinity and help-seeking among men with depression: A qualitative study. Front Psychiatry. (2020) 11:599039. doi: 10.3389/fpsyt.2020.599039 33329149 PMC7732518

[8] Augsberger A Yeung A Dougher M Hahm HC. Factors influencing the underutilization of mental health services among Asian American women with a history of depression and suicide. BMC Health Serv Res. (2015) 15:542. doi: 10.1186/s12913-015-1191-7 26645481 PMC4673784

[9] Lin KM Inui TS Kleinman AM Womack WM. Sociocultural determinants of the help-seeking behavior of patients with mental illness. J Nerv Ment Dis. (1982) 170:78–85. doi: 10.1097/00005053-198202000-00003 7057173

[10] Koopmans GT Lamers LM. Gender and health care utilization: The role of mental distress and help-seeking propensity. Soc Sci Med. (2007) 64:1216–30. doi: 10.1016/j.socscimed.2006.11.018 17194514

[11] Arnault DS. Cultural Determinants of Help Seeking: A model for research and practice. Res Theory Nurs Pract. (2009) 23:259–78. doi: 10.1891/1541-6577.23.4.259 PMC2796597 19999745

[12] Nadler A. Personality and Help Seeking. In: Pierce GR Lakey B Sarason IG Sarason BR, editors. Sourcebook of Social Support and Personality. Springer US, Boston, MA (1997). p. 379–407. doi: 10.1007/978-1-4899-1843-7_17

[13] Riddle MA Maher BS Wang Y Grados M Bienvenu OJ Goes FS. OBSESSIVE-COMPULSIVE PERSONALITY DISORDER: EVIDENCE FOR TWO DIMENSIONS: research article: obsessive-compulsive personality disorder. Depress Anxiety. (2016) 33:128–35. doi: 10.1002/da.2016.33.issue-2 26594839

[14] Perry JD Perry JC. Conflicts, defenses and the stability of narcissistic personality features. Psychiatry. (2004) 67(4):310–30. doi: 10.1521/psyc.67.4.310.56570 15801375

[15] Tharp JA Johnson SL Dev A. Transdiagnostic approach to the dominance behavioral system. Pers Individ Differ. (2021) 176:110778. doi: 10.1016/j.paid.2021.110778 PMC7978407 33746322

[16] ten Have M Oldehinkel A Vollebergh W Ormel J. Does neuroticism explain variations in care service use for mental health problems in the general population? Soc Psychiatry Psychiatr Epidemiol. (2005) 40:425–31. doi: 10.1007/s00127-005-0916-z 16003591

[17] Sansone RA Farukhi S Wiederman MW. Utilization of primary care physicians in borderline personality. Gen Hosp Psychiatry. (2011) 33:343–6. doi: 10.1016/j.genhosppsych.2011.04.006 21762830

[18] Hassan N Sumardi NA Aziz RA. The influence of personality traits on communication competence. Int J Acad Res Bus Soc Sci. (2019) 9:493–505. doi: 10.6007/IJARBSS/v9-i13/6999

[19] Amirkhan JH Risinger RT Swickert RJ. Extraversion: A “Hidden” Personality factor in coping? J Pers. (1995) 63:189–212. doi: 10.1111/j.1467-6494.1995.tb00807.x 7782992

[20] Samuel DB Bucher MA Suzuki T. A preliminary probe of personality predicting psychotherapy outcomes: perspectives from therapists and their clients. Psychopathology. (2018) 51:122–9. doi: 10.1159/000487362 29635236

[21] Patterson C Perlman D Moxham L Burns S. Do help-seeking behaviors influence the recovery of people with mental illness? J Psychosoc Nurs Ment Health Serv. (2019) 57:33–8. doi: 10.3928/02793695-20190920-03 31566704

[22] Welsh J Korda RJ Banks E Strazdins L Joshy G Butterworth P. Identifying long-term psychological distress from single measures: evidence from a nationally representative longitudinal survey of the Australian population. BMC Med Res Methodol. (2020) 20:55. doi: 10.1186/s12874-020-00938-8 32138694 PMC7059354

[23] Tanji F Tomata Y Zhang S Otsuka T Tsuji I. Psychological distress and completed suicide in Japan: A comparison of the impact of moderate and severe psychological distress. Prev Med. (2018) 116:99–103. doi: 10.1016/j.ypmed.2018.09.007 30219687

[24] Rai D Kosidou K Lundberg M Araya R Lewis G Magnusson C. Psychological distress and risk of long-term disability: population-based longitudinal study. J Epidemiol Community Health. (2012) 66:586–92. doi: 10.1136/jech.2010.119644 21422028

[25] Gulliver A Griffiths KM Christensen H Brewer JL. A systematic review of help-seeking interventions for depression, anxiety and general psychological distress. BMC Psychiatry. (2012) 12:81. doi: 10.1186/1471-244X-12-81 22799879 PMC3464688

[26] Conrod PJ Castellanos-Ryan N Mackie C. Long-term effects of a personality-targeted intervention to reduce alcohol use in adolescents. J Consult Clin Psychol. (2011) 79:296–306. doi: 10.1037/a0022997 21500886

[27] O’Leary-Barrett M Topper L Al-Khudhairy N Pihl RO Castellanos-Ryan N Mackie CJ. Two-year impact of personality-targeted, teacher-delivered interventions on youth internalizing and externalizing problems: A cluster-randomized trial. J Am Acad Child Adolesc Psychiatry. (2013) 52:911–20. doi: 10.1016/j.jaac.2013.05.020 23972693

[28] Page MJ McKenzie JE Bossuyt PM Boutron I Hoffmann TC Mulrow CD. The PRISMA 2020 statement: an updated guideline for reporting systematic reviews. BMJ. (2021) 372:n71. doi: 10.1136/bmj.n71 33782057 PMC8005924

[29] The role of personality traits in help-seeking for psychological distress - PROSPERO Protocol (2023). Available online at: https://www.crd.york.ac.uk/prospero/display_record.php?RecordID=388285 (Accessed February 27, 2024).

[30] Evans SC Amaro CM Herbert R Blossom JB Roberts MC. Are you gonna publish that?” Peer-reviewed publication outcomes of doctoral dissertations in psychology. PloS One. (2018) 13:e0192219. doi: 10.1371/journal.pone.0192219 29444130 PMC5812605

[31] American Psychological Association (APA). Personality (2018). Available online at: http://www.apa.org/topics/personality/index.aspx (Accessed February 21, 2024).

[32] American Psychiatric Association. Diagnostic and statistical manual of mental disorders (DSM-5®). Washington DC, USA: American Psychiatric Association (2013).

[33] ICD-11 (2024). Available online at: https://icd-who-int.libproxy1.nus.edu.sg/en (Accessed February 22, 2024).

[34] Digman JM. Personality structure: Emergence of the five-factor model. Annu Rev Psychol. (1990) 41:417–40. doi: 10.1146/annurev.ps.41.020190.002221

[35] Costa PT McCrae RR. Manual for the revised NEO personality inventory (NEO-PI-R) and NEO five-factor inventory (NEO-FFI) . Odessa, FL, USA: Psychological Assessment Resources. (1992).

[36] Shedler J Westen D. Dimensions of personality pathology: an alternative to the five-factor model. Am J Psychiatry. (2004) 161:1743–54. doi: 10.1176/ajp.161.10.1743 15465966

[37] McWilliams LA Cox BJ Enns MW Clara IP. Personality correlates of outpatient mental health service utilization: findings from the US national comorbidity survey. Soc Psychiatry Psychiatr Epidemiol. (2006) 41:357–63. doi: 10.1007/s00127-006-0040-8 16565922

[38] Viertiö S Kiviruusu O Piirtola M Kaprio J Korhonen T Marttunen M. Factors contributing to psychological distress in the working population, with a special reference to gender difference. BMC Public Health. (2021) 21:611. doi: 10.1186/s12889-021-10560-y 33781240 PMC8006634

[39] Psychological distress (2024). Available online at: https://dictionary.apa.org/ (Accessed February 22, 2024).

[40] Bramer WM Rethlefsen ML Kleijnen J Franco OH. Optimal database combinations for literature searches in systematic reviews: a prospective exploratory study. Syst Rev. (2017) 6:245. doi: 10.1186/s13643-017-0644-y 29208034 PMC5718002

[41] Gotschall T. EndNote 20 desktop version. J Med Libr Assoc. (2021) 109:520–2. doi: 10.5195/jmla.2021.1260 PMC8485940 34629985

[42] Bramer WM Giustini D de Jonge GB Holland L Bekhuis T. De-duplication of database search results for systematic reviews in EndNote. J Med Libr Assoc JMLA. (2016) 104:240. doi: 10.3163/1536-5050.104.3.014 27366130 PMC4915647

[43] Ouzzani M Hammady H Fedorowicz Z Elmagarmid A. Rayyan—a web and mobile app for systematic reviews. Syst Rev. (2016) 5:210. doi: 10.1186/s13643-016-0384-4 27919275 PMC5139140

[44] The Ottawa Hospital. The Newcastle-Ottawa Scale (NOS) for assessing the quality of nonrandomised studies in meta-analyses (2024). Available online at: https://www.ohri.ca/programs/clinical_epidemiology/oxford.asp (Accessed February 22, 2024).

[45] Lo CKL Mertz D Loeb M. Newcastle-Ottawa Scale: comparing reviewers’ to authors’ assessments. BMC Med Res Methodol. (2014) 14:45. doi: 10.1186/1471-2288-14-45 24690082 PMC4021422

[46] Herzog R Álvarez-Pasquin MJ Díaz C Del Barrio JL Estrada JM Gil Á. Are healthcare workers’ intentions to vaccinate related to their knowledge, beliefs and attitudes? a systematic review. BMC Public Health. (2013) 13:154. doi: 10.1186/1471-2458-13-154 23421987 PMC3602084

[47] Braham CA White PJ Arinaminpathy N. Management of tuberculosis by healthcare practitioners in Pakistan: A systematic review. Singh JA editor PloS One. (2018) 13:e0199413. doi: 10.1371/journal.pone.0199413 PMC6013248 29928031

[48] Fischer EH Turner JI. Orientations to seeking professional help: development and research utility of an attitude scale. J Consult Clin Psychol. (1970) 35:79. doi: 10.1037/h0029636 5487612

[49] Peterson RA Brown SP. On the use of beta coefficients in meta-analysis. J Appl Psychol. (2005) 90:175. doi: 10.1037/0021-9010.90.1.175 15641898

[50] Roth PL Le H Oh I Van Iddekinge CH Bobko P. Using beta coefficients to impute missing correlation coefficients in meta-analysis research: reasons for caution. J Appl Psychol. (2018) 103(6):644–58. doi: 10.1037/apl0000293 29369653

[51] Arbisi PA Rusch L Polusny MA Thuras P Erbes CR. Does cynicism play a role in failure to obtain needed care? Mental health service utilization among returning U.S. National Guard soldiers. Psychol Assess. (2013) 25:991–6. doi: 10.1037/a0032225 23544401

[52] Atik G Yalçin Y. Help-seeking attitudes of university students: the role of personality traits and demographic factors. South Afr J Psychol. (2011) 41:328–38. doi: 10.1177/008124631104100307

[53] Billingsley KD. Predictors of attitudes toward seeking professional psychological help: A survey of older and younger adults. United States – Ohio: The University of Akron (1999). Available at: https://www.proquest.com/docview/304410620/abstract/F94D6E77FD43485EPQ/1 (Accessed February 23, 2024).

[54] Blanch S Barkus E. Schizotypy and help-seeking for anxiety. Early Interv Psychiatry. (2021) 15:1433–6. doi: 10.1111/eip.13079 33242926

[55] Boerema AM Kleiboer A Beekman ATF Van Zoonen K Dijkshoorn H Cuijpers P. Determinants of help-seeking behavior in depression: a cross-sectional study. BMC Psychiatry. (2016) 16:78. doi: 10.1186/s12888-016-0790-0 27009062 PMC4806501

[56] Broadbear JH Dwyer J Bugeja L Rao S. Coroners’ investigations of suicide in Australia: The hidden toll of borderline personality disorder. J Psychiatr Res. (2020) 129:241–9. doi: 10.1016/j.jpsychires.2020.07.007 32823217

[57] Cole BP. An Exploration of Men’s Attitudes Regarding Depression and Help-Seeking. United States – Nebraska: The University of Nebraska - Lincoln (2014). Available at: https://www.proquest.com/docview/1352754784/abstract/8EF0CF6E9414525PQ/1 (Accessed February 23, 2024).

[58] Cortese JR. Gender role conflict, personality, and help -seeking in adult men. United States – California: University of Southern California (2004). Available at: https://www.proquest.com/docview/305305367/abstract/744875B2B3A047D8PQ/1 (Accessed February 23, 2024).

[59] Cuijpers P Steunenberg B Van Straten A. Actions taken to cope with depressed mood: The role of personality traits. Aging Ment Health. (2007) 11:457–63. doi: 10.1080/13607860601086496 17612810

[60] Dalum HS Tyssen R Moum T Thoresen M Hem E. Professional help-seeking behaviour for mental health problems among veterinarians in Norway: a nationwide, cross-sectional study (The NORVET study). BMC Public Health. (2022) 22:1308. doi: 10.1186/s12889-022-13710-y 35799295 PMC9263054

[61] Dang SS Quesnel DA Hewitt PL Flett GL Deng X. Perfectionistic traits and self-presentation are associated with negative attitudes and concerns about seeking professional psychological help. Clin Psychol Psychother. (2020) 27:621–9. doi: 10.1002/cpp.v27.5 32222088

[62] Drapeau CW Cerel J Moore M. How personality, coping styles, and perceived closeness influence help-seeking attitudes in suicide-bereaved adults. Death Stud. (2016) 40:165–71. doi: 10.1080/07481187.2015.1107660 26745343

[63] Eurelings-Bontekoe EH van der Slikke M Verschuur MJ. Psychological distress, depressive symptomatology, coping and DSM-III-R/ICD-10 personality disorders a study among primary mental health care patients. Pers Individ Differ. (1997) 23:407–17. doi: 10.1016/S0191-8869(97)80006-2

[64] Fekih-Romdhane F Amri A Cheour M. Suicidal ideation, suicide literacy and stigma, disclosure expectations and attitudes toward help-seeking among university students: The impact of schizotypal personality traits. Early Interv Psychiatry. (2022) 16:659–69. doi: 10.1111/eip.13211 34477298

[65] Gormley N O’Leary D. Time to initial medical presentation in a first-admission group with depression. Acta Psychiatr Scand. (1998) 97:166–7. doi: 10.1111/j.1600-0447.1998.tb09981.x 9517913

[66] Hatchett GT Park HL. Reexamination of the five-factor model and college students’ Treatment-seeking attitudes. J Couns Dev. (2019) 97:140–7. doi: 10.1002/jcad.2019.97.issue-2

[67] Hayslip B Jr Maiden RJ Thomison NL Temple JR. Mental health attitudes among rural and urban older adults. Clin Gerontol. (2010) 33:316–31. doi: 10.1080/07317115.2010.503557

[68] Hyland P Boduszek D Dhingra K Shevlin M Maguire R Morley K. A test of the inventory of attitudes towards seeking mental health services. Br J Guid Couns. (2015) 43:397–412. doi: 10.1080/03069885.2014.963510

[69] Ingram PB IV Lichtenberg JW Clarke E. Self-stigma, personality traits, and willingness to seek treatment in a community sample. Psychol Serv. (2016) 13:300. doi: 10.1037/ser0000086 27253319

[70] Iza M Olfson M Vermes D Hoffer M Wang S Blanco C. Probability and predictors of first treatment contact for anxiety disorders in the United States: analysis of data from the National Epidemiologic Survey on Alcohol and Related Conditions (NESARC). J Clin Psychiatry. (2013) 74:15656. doi: 10.4088/JCP.13m08361 24330896

[71] Joyce N. An empirical examination of the influence of personality, gender role conflict, and self-stigma on attitudes and intentions to seek online counseling in college students. United States – Ohio: The University of Akron (2013). Available at: https://www.proquest.com/docview/1086348966/abstract/EA7574FEA8EA4279PQ/1 (Accessed February 23, 2024).

[72] Kakhnovets R. Relationships among personality, expectations about counseling, and help-seeking attitudes. J Couns Dev. (2011) 89:11–9. doi: 10.1002/j.1556-6678.2011.tb00056.x

[73] Kessler EM Agines S Bowen CE. Attitudes towards seeking mental health services among older adults: Personal and contextual correlates. Aging Ment Health. (2015) 19:182–91. doi: 10.1080/13607863.2014.920300 24898327

[74] McCrae RR Costa PT. Personality, coping, and coping effectiveness in an adult sample. J Pers. (1986) 54:385–404. doi: 10.1111/j.1467-6494.1986.tb00401.x

[75] Michal M Wiltink J Grande G Beutel ME Brähler E. Type D personality is independently associated with major psychosocial stressors and increased health care utilization in the general population. J Affect Disord. (2011) 134:396–403. doi: 10.1016/j.jad.2011.05.033 21663973

[76] Miller PJ. Personality as a potential moderator of the relationship between stigma and help -seeking. United States – Iowa: Iowa State University (2010). Available at: https://www.proquest.com/docview/304902053/abstract/FB58F14BF64841EEPQ/1 (Accessed February 23, 2024).

[77] Minge MR Bowman TF. Personality differences among nonclients and vocational-educational and personal counseling clients. J Couns Psychol. (1967) 14:137. doi: 10.1037/h0024422

[78] O’connor PJ Martin B Weeks CS Ong L. Factors that influence young people’s mental health help-seeking behaviour: a study based on the Health Belief Model. J Adv Nurs. (2014) 70:2577–87. doi: 10.1111/jan.12423 24720449

[79] Oluyinka O. Psychological predictors of attitude towards seeking professional psychological help in a Nigerian university student population. South Afr J Psychol. (2011) 41:310–27. doi: 10.1177/008124631104100306

[80] Park S Lee Y Seong SJ Chang SM Lee JY Hahm BJ. A cross-sectional study about associations between personality characteristics and mental health service utilization in a Korean national community sample of adults with psychiatric disorders. BMC Psychiatry. (2017) 17:170. doi: 10.1186/s12888-017-1322-2 28476104 PMC5420157

[81] Pugh JS. Help seeking and personality among college students. United States – Illinois: Southern Illinois University at Carbondale (2002). Available at: https://www.proquest.com/docview/304623488/abstract/B649FADB558945C2PQ/1 (Accessed February 23, 2024).

[82] Puma LM. The relative contribution of knowledge of counseling services, personality style, and psychological distress on attitudes toward seeking professional help: A test of three theoretical models. United States – Ohio: The University of Akron (1996). Available at: https://www.proquest.com/docview/304231646/abstract/3BAEEC7AA663428CPQ/1 (Accessed February 23, 2024).

[83] Rankine JL. Major Predictors of Willingness to Seek Mental Health Services: Impact of Personality, Age, Gender, and Stigma. United States – Florida: Keiser University (2021). Available at: https://www.proquest.com/docview/2528547996/abstract/735E0DDA69A0491BPQ/1 (Accessed February 23, 2024).

[84] Rim Y. Ways of coping, personality, age, sex and family structural variables. Pers Individ Differ. (1986) 7:113–6. doi: 10.1016/0191-8869(86)90115-7

[85] Samuel R Kamenetsky SB. Help-Seeking Preferences and Factors Associated with Attitudes toward Seeking Mental Health Services among First-Year Undergraduates. Can J High Educ. (2022) 52:30–50. doi: 10.47678/cjhe.v52i1.189245

[86] Schomerus G Appel K Meffert PJ Luppa M Andersen RM Grabe HJ. Personality-related factors as predictors of help-seeking for depression: a population-based study applying the behavioral model of health services use. Soc Psychiatry Psychiatr Epidemiol. (2013) 48:1809–17. doi: 10.1007/s00127-012-0643-1 23266663

[87] Shahaf-Oren B Madan I Henderson C. A lot of medical students, their biggest fear is failing at being seen to be a functional human”: disclosure and help-seeking decisions by medical students with health problems. BMC Med Educ. (2021) 21:599. doi: 10.1186/s12909-021-03032-9 34865636 PMC8645095

[88] Svanborg C Rosso MS Lützen K Wistedt AÅ Bäärnhielm S. Barriers in the help-seeking process: A multiple-case study of early-onset dysthymia in Sweden. Nord J Psychiatry. (2008) 62:346–53. doi: 10.1080/08039480801959315 18752107

[89] Tomko RL Trull TJ Wood PK Sher KJ. Characteristics of borderline personality disorder in a community sample: comorbidity, treatment utilization, and general functioning. J Pers Disord. (2014) 28:734–50. doi: 10.1521/pedi_2012_26_093 PMC3864176 25248122

[90] Tyssen R Røvik JO Vaglum P Grønvold NT Ekeberg Ø. Help-seeking for mental health problems among young physicians: is it the most ill that seeks help? A longitudinal and nationwide study. Soc Psychiatry Psychiatr Epidemiol. (2004) 39:989–93. doi: 10.1007/s00127-004-0831-8 15583907

[91] Ullrich S Coid J. Antisocial personality disorder: Co-morbid Axis I mental disorders and health service use among a national household population. Pers Ment Health. (2009) 3:151–64. doi: 10.1002/pmh.v3:3

[92] Valipay S Parikh M Desai M Nathametha B. A study of factors affecting help-seeking behavior in major depressive disorder. Ann Indian Psychiatry. (2019) 3:148–8. doi: 10.4103/aip.aip_30_19

[93] Van Zoonen K Kleiboer A Beekman ATF Smit JH Boerema AM Cuijpers P. Reasons and determinants of help-seeking in people with a subclinical depression. J Affect Disord. (2015) 173:105–12. doi: 10.1016/j.jad.2014.10.062 25462403

[94] Yasmeen S Tangney JP Stuewig JB Hocter C Weimer L. The implications of borderline personality features for jail inmates’ institutional misconduct and treatment-seeking. Pers Disord Theory Res Treat. (2022) 13:505–15. doi: 10.1037/per0000518 34780233

[95] Yelpaze İ Ceyhan AA. The prediction of personality, culture and coping strategies on university students’ psychological help seeking attitudes. Turk J Educ. (2020) 9:134–53. doi: 10.19128/turje.611402

[96] Yi SH. Help-seeking behavior of Korean-Americans: Implications for psychological adjustment. United States – California: University of California, Los Angeles (1998). Available at: https://www.proquest.com/docview/304351944/abstract/8FF6C1B1799E44D7PQ/1 (Accessed February 23, 2024).

[97] Maier W Lichtermann D Oehrlein A Fickinger M. Depression in the community: a comparison of treated and non-treated cases in two non-referred samples. Psychopharmacol (Berl). (1992) 106:S79–81. doi: 10.1007/BF02246242 1546148

[98] Eysenck HJ. Psychophysiology and Personality: Extraversion, Neuroticism and Psychoticism. In: Individual Differences and Psychopathology. Amsterdam, Netherlands: Elsevier (1983). p. 13–30. Available at: https://linkinghub.elsevier.com/retrieve/pii/B9780122739033500079 (Accessed February 25, 2024).

[99] Costa PT McCrae RR. Primary traits of Eysenck’s P-E-N system: Three- and five-factor solutions. J Pers Soc Psychol. (1995) 69:308–17. doi: 10.1037/0022-3514.69.2.308 7643307

[100] Fischer EH Farina A. Attitudes toward seeking professional psychologial help: A shortened form and considerations for research. J Coll Stud Dev. (1995) 36(4):368–73. doi: 10.1037/t05375-000

[101] Mackenzie CS El-Gabalawy R Chou KL Sareen J. Prevalence and predictors of persistent versus remitting mood, anxiety, and substance disorders in a national sample of older adults. J Geriatr Psychiatry. (2014) 22:854–65. doi: 10.1016/j.jagp.2013.02.007 PMC4284958 23800537

[102] ICD-10 Version (2019). Available online at: https://icd-who-int.libproxy1.nus.edu.sg/browse10/2019/en/F60-F69 (Accessed February 25, 2024).

[103] Kim LE Chen L MacCann C Karlov L Kleitman S. Evidence for three factors of perfectionism: Perfectionistic Strivings, Order, and Perfectionistic Concerns. Pers Individ Differ. (2015) 84:16–22. doi: 10.1016/j.paid.2015.01.033

[104] Costa PT McCrae RR. Revised NEO personality inventory. Lutz, FL: Psychological Assessment Resources (1992). p. 101.

[105] Sakado K Sato T Uehara T Sato S Sakado M Kumagai K. Evaluating the diagnostic specificity of the Munich personality test dimensions in major depression. J Affect Disord. (1997) 43:187–94. doi: 10.1016/S0165-0327(97)01434-1 9186789

[106] Hummelen B Wilberg T Pedersen G Karterud S. The quality of the DSM-IV obsessive-compulsive personality disorder construct as a prototype category. J Nerv Ment Dis. (2008) 196:446–55. doi: 10.1097/NMD.0b013e3181775a4e 18552621

[107] Grilo CM Skodol AE Gunderson JG Sanislow CA Stout RL Shea MT. Longitudinal diagnostic efficiency of DSM-IV criteria for obsessive-compulsive personality disorder: a 2-year prospective study. Acta Psychiatr Scand. (2004) 110:64–8. doi: 10.1111/j.1600-0447.2004.00311.x 15180781

[108] Nestadt G Romanoski AJ Chahal R Merchant A Folstein MF Gruenberg EM. An epidemiological study of histrionic personality disorder. Psychol Med. (1990) 20:413–22. doi: 10.1017/S0033291700017724 2356266

[109] Blay M Cham MA Duarte M Ronningstam E. Association between pathological narcissism and emotion dysregulation: A systematic review. Psychopathology. (2024) 57:297–317. doi: 10.1159/000538546 38870915

[110] Ene C Rîndaşu C Ionescu D. Patients’ personality in disease self-management. A self-determination perspective. Curr Psychol. (2023) 42:9618–26. doi: 10.1007/s12144-021-02240-2

[111] Auerbach RP Abela JRZ Ringo Ho MH. Responding to symptoms of depression and anxiety: Emotion regulation, neuroticism, and engagement in risky behaviors. Behav Res Ther. (2007) 45:2182–91. doi: 10.1016/j.brat.2006.11.002 17181999

[112] De Fruyt F Denollet J. Type D personality: A five-factor model perspective. Psychol Health. (2002) 17:671–83. doi: 10.1080/08870440290025858

[113] Torgersen S Alnæs R. Localizing DSM-III personality disorders in a three-dimensional structural space. J Pers Disord. (1989) 3:274–81. doi: 10.1521/pedi.1989.3.4.274

[114] Torgersen S. Personlighet og personlighetsforstyrrelser. Oslo, Norway: Gyldendal akademisk (2008).

[115] Aldinger M Stopsack M Ulrich I Appel K Reinelt E Wolff S. Neuroticism developmental courses - implications for depression, anxiety and everyday emotional experience; a prospective study from adolescence to young adulthood. BMC Psychiatry. (2014) 14:210. doi: 10.1186/s12888-014-0210-2 25207861 PMC4158099

[116] Skodol AE Grilo CM Keyes KM Geier T Grant BF Hasin DS. Relationship of personality disorders to the course of major depressive disorder in a nationally representative sample. Am J Psychiatry. (2011) 168:257–64. doi: 10.1176/appi.ajp.2010.10050695 PMC3202962 21245088

[117] Wang Y Shi HS Liu WH Xie DJ Geng FL Yan C. Trajectories of schizotypy and their emotional and social functioning: An 18-month follow-up study. Schizophr Res. (2018) 193:384–90. doi: 10.1016/j.schres.2017.07.038 28751128

[118] Ng W Diener E. Personality differences in emotions. J Individ Differ. (2009) 30:100–6. doi: 10.1027/1614-0001.30.2.100

[119] Miano A Grosselli L Roepke S Dziobek I. Emotional dysregulation in borderline personality disorder and its influence on communication behavior and feelings in romantic relationships. Behav Res Ther. (2017) 95:148–57. doi: 10.1016/j.brat.2017.06.002 28646782

[120] Karterud S Pedersen G Bjordal E Brabrand J Friis S Haaseth O. Day treatment of patients with personality disorders: experiences from a Norwegian treatment research network. J Pers Disord. (2003) 17:243–62. doi: 10.1521/pedi.17.3.243.22151 12839103

[121] Harwood D Hawton K Hope T Jacoby R. Psychiatric disorder and personality factors associated with suicide in older people: a descriptive and case-control study. Int J Geriatr Psychiatry. (2001) 16:155–65. doi: 10.1002/1099-1166(200102)16:2<155::AID-GPS289>3.0.CO;2-0 11241720

[122] Conwell Y Van Orden K Caine ED. Suicide in older adults. Psychiatr Clin North Am. (2011) 34:451–68. doi: 10.1016/j.psc.2011.02.002 PMC3107573 21536168

[123] Waern M Beskow J Runeson B Skoog I. Suicidal feelings in the last year of life in elderly people who commit suicide. Lancet. (1999) 354:917–8. doi: 10.1016/S0140-6736(99)93099-4 10489955

[124] Quilty LC De Fruyt F Rolland JP Kennedy SH Rouillon P Bagby RM. Dimensional personality traits and treatment outcome in patients with major depressive disorder. J Affect Disord. (2008) 108:241–50. doi: 10.1016/j.jad.2007.10.022 18067975

[125] Mojtabai R Evans-Lacko S Schomerus G Thornicroft G. Attitudes toward mental health help seeking as predictors of future help-seeking behavior and use of mental health treatments. Psychiatr Serv. (2016) 67:650–7. doi: 10.1176/appi.ps.201500164 26876662

[126] Delgadillo J Lutz W. A development pathway towards precision mental health care. JAMA Psychiatry. (2020) 77:889. doi: 10.1001/jamapsychiatry.2020.1048 32459326

[127] Roberts RO Bergstralh EJ Schmidt L Jacobsen SJ. Comparison of self-reported and medical record health care utilization measures. J Clin Epidemiol. (1996) 49:989–95. doi: 10.1016/0895-4356(96)00143-6 8780606

