# Supplementary material for: Personality and help-seeking for psychological distress: a systematic review and meta-analysis
Source: Front Psychiatry. 2025 Jan 23;15:1405167. doi: 10.3389/fpsyt.2024.1405167 (PMC11798934; doi:10.3389/fpsyt.2024.1405167)

**Supplement**

**Table S1 - Search strategy**

|  | | **Concept 1 /Population/Problem** | **Concept2**  **/Intervention/Exposure** |
| --- | --- | --- | --- |
| **Key concepts**  *Identify the key concepts based on your research topic.* | | **Personality** | **Help-seeking** |
| **Free text terms / natural language terms**  (synonyms, UK/US terminology, medical/layman's terms, acronyms/abbreviations, drug brands, more narrow search terms)  *List down your keywords for each concept.* | | personalit*[Title/Abstract]  OR  trait*[Title/Abstract]  OR  neuroticism[Title/Abstract]  OR  extraversion[Title/Abstract]  OR  conscientiousness[Title/Abstract] OR  openness[Title/Abstract]  OR  agreeableness[Title/Abstract] | seek help[Title/Abstract]  OR  help seek*[Title/Abstract]  OR  seek care[Title/Abstract]  OR  care seek*[Title/Abstract]  OR  treatment seek*[Title/Abstract]  OR  seeking treatment[Title/Abstract]  OR  self-referral[Title/Abstract] |
| **Medline (PubMed) / Cochrane Library)** | **Controlled vocabulary** **terms**  MeSH terms | Personality[Mesh]  Personality Disorder[Mesh] | Help-Seeking Behavior[Mesh] |

**Table S2 – Risk of bias assessment of all included records**

|  | **[I] Representa-tiveness of the sample**  **(max = 1 point)** | **[II]**  **Sample size**  **(max = 1 point)** | **[III]**  **Non-respondents**  **(max = 1 point)** | **[IV] Ascertainment of the exposure**  **(max = 2 points)** | **[V] Comparability**  **(max = 2 points)** | **[VI-1] Outcome reliability**  **(max = 2 points)** | **[VI-2] Statistical test**    **(max = 1 point)** | **GRAND TOTAL** | **Rating** |
| --- | --- | --- | --- | --- | --- | --- | --- | --- | --- |
| Arbisi, 2013 | 1 | 1 | 1 | 1 | 2 | 1 | 1 | 8 | high |
| Atik, 2011 | 0 | 1 | 0 | 2 | 1 | 1 | 1 | 6 | moderate |
| Billingsley, 1999 (T) | 0 | 1 | 0 | 2 | 2 | 1 | 0 | 6 | moderate |
| Blanch, 2021 | 1 | 1 | 0 | 2 | 0 | 1 | 0 | 5 | moderate |
| Boerema, 2016 | 1 | 0 | 0 | 2 | 2 | 1 | 1 | 7 | high |
| Broadbear, 2020 | 1 | 1 | 1 | 2 | 1 | 2 | 0 | 8 | high |
| Cole, 2014 (T) | 0 | 1 | 0 | 1 | 1 | 1 | 0 | 4 | moderate |
| Cortese, 2004 (T) | 1 | 1 | 1 | 2 | 1 | 1 | 0 | 7 | high |
| Cuijpers, 2007 | 1 | 1 | 0 | 2 | 2 | 1 | 0 | 7 | high |
| Dalum, 2022 | 1 | 1 | 1 | 1 | 2 | 1 | 1 | 8 | high |
| Dang, 2020 | 1 | 0 | 0 | 2 | 0 | 1 | 0 | 4 | moderate |
| Drapeau, 2016 | 1 | 1 | 1 | 2 | 1 | 1 | 1 | 8 | high |
| Eurelings-Bontekoe, 1997 | 0 | 0 | 1 | 2 | 1 | 1 | 0 | 5 | moderate |
| Fekih-Romdhane, 2021 | 1 | 1 | 1 | 2 | 2 | 1 | 0 | 8 | high |
| Gormley, 1998 | 1 | 0 | 1 | 2 | 0 | 2 | 1 | 7 | high |
| Hatchett, 2019 | 1 | 1 | 0 | 2 | 2 | 1 | 1 | 8 | high |
| Hayslip, 2010 | 0 | 0 | 0 | 2 | 0 | 1 | 0 | 3 | low |
| Hyland, 2015 | 0 | 0 | 0 | 2 | 1 | 1 | 1 | 5 | moderate |
| Ingram, 2016 | 1 | 0 | 0 | 2 | 1 | 1 | 1 | 6 | moderate |
| Iza, 2013 | 1 | 1 | 1 | 2 | 2 | 1 | 1 | 9 | high |
| Joyce, 2013 (T) | 1 | 1 | 0 | 2 | 1 | 1 | 1 | 7 | high |
| Kakhnovets, 2011 | 1 | 1 | 0 | 2 | 1 | 1 | 1 | 7 | high |
| Kessler, 2015 | 0 | 0 | 0 | 2 | 1 | 1 | 1 | 5 | moderate |
| Maier, 1992 | 1 | 1 | 0 | 2 | 1 | 1 | 0 | 6 | moderate |
| McCrae, 1986 (2 studies) | 0 | 0 | 0 | 2 | 0 | 1 | 1 | 4 | moderate |
| Michal, 2011 | 1 | 1 | 1 | 2 | 2 | 1 | 1 | 9 | high |
| Miller, 2010 (T) | 1 | 1 | 1 | 2 | 1 | 1 | 1 | 8 | high |
| Minge, 1967 | 0 | 0 | 0 | 2 | 0 | 2 | 0 | 4 | moderate |
| O'Connor, 2014 | 1 | 0 | 1 | 2 | 1 | 1 | 0 | 6 | moderate |
| Oluyinka, 2011 | 1 | 1 | 1 | 2 | 1 | 1 | 1 | 8 | high |
| Park, 2017 | 1 | 1 | 0 | 2 | 2 | 1 | 1 | 8 | high |
| Pugh, 2002 (T) | 1 | 0 | 0 | 2 | 1 | 1 | 0 | 5 | moderate |
| Puma, 1996 (T) | 1 | 1 | 0 | 2 | 2 | 1 | 0 | 7 | high |
| Rankine, 2021 (T) | 0 | 1 | 0 | 2 | 1 | 1 | 1 | 6 | moderate |
| Rim, 1986 | 0 | 0 | 0 | 2 | 0 | 1 | 0 | 3 | low |
| Samuel, 2022 | 0 | 0 | 0 | 2 | 1 | 1 | 1 | 5 | moderate |
| Schomerus, 2013 | 1 | 0 | 1 | 2 | 2 | 1 | 1 | 8 | high |
| Shahaf, 2021 | Not applicable (qualitative study) | | | | | | | | |
| Svanborg, 2008 | Not applicable (qualitative study) | | | | | | | | |
| Tomko, 2014 | 1 | 1 | 0 | 2 | 0 | 1 | 1 | 6 | moderate |
| Tyssen, 2004 | 1 | 1 | 1 | 2 | 2 | 1 | 1 | 9 | high |
| Ullrich, 2009 | 1 | 1 | 1 | 2 | 2 | 1 | 1 | 9 | high |
| Valipay, 2019 | 1 | 0 | 0 | 2 | 0 | 1 | 1 | 5 | moderate |
| van Zoonen, 2015 | 1 | 0 | 1 | 2 | 1 | 1 | 1 | 7 | high |
| Yasmeen, 2022 | 1 | 1 | 1 | 2 | 1 | 2 | 1 | 9 | high |
| Yelpaze, 2020 | 0 | 1 | 0 | 1 | 1 | 1 | 1 | 5 | moderate |
| Yi, 1998 (T) | 0 | 0 | 0 | 2 | 1 | 1 | 0 | 4 | moderate |

Legend: (T), doctoral thesis; yellow cells mark categories where the study lost one point, orange cells mark categories where the study lost two points; green and red cells in the last column indicate high evidence quality and low evidence quality, respectively.

**Figure S1 – Sensitivity meta-analysis including only studies that reported correlation coefficients for the associations of interest**


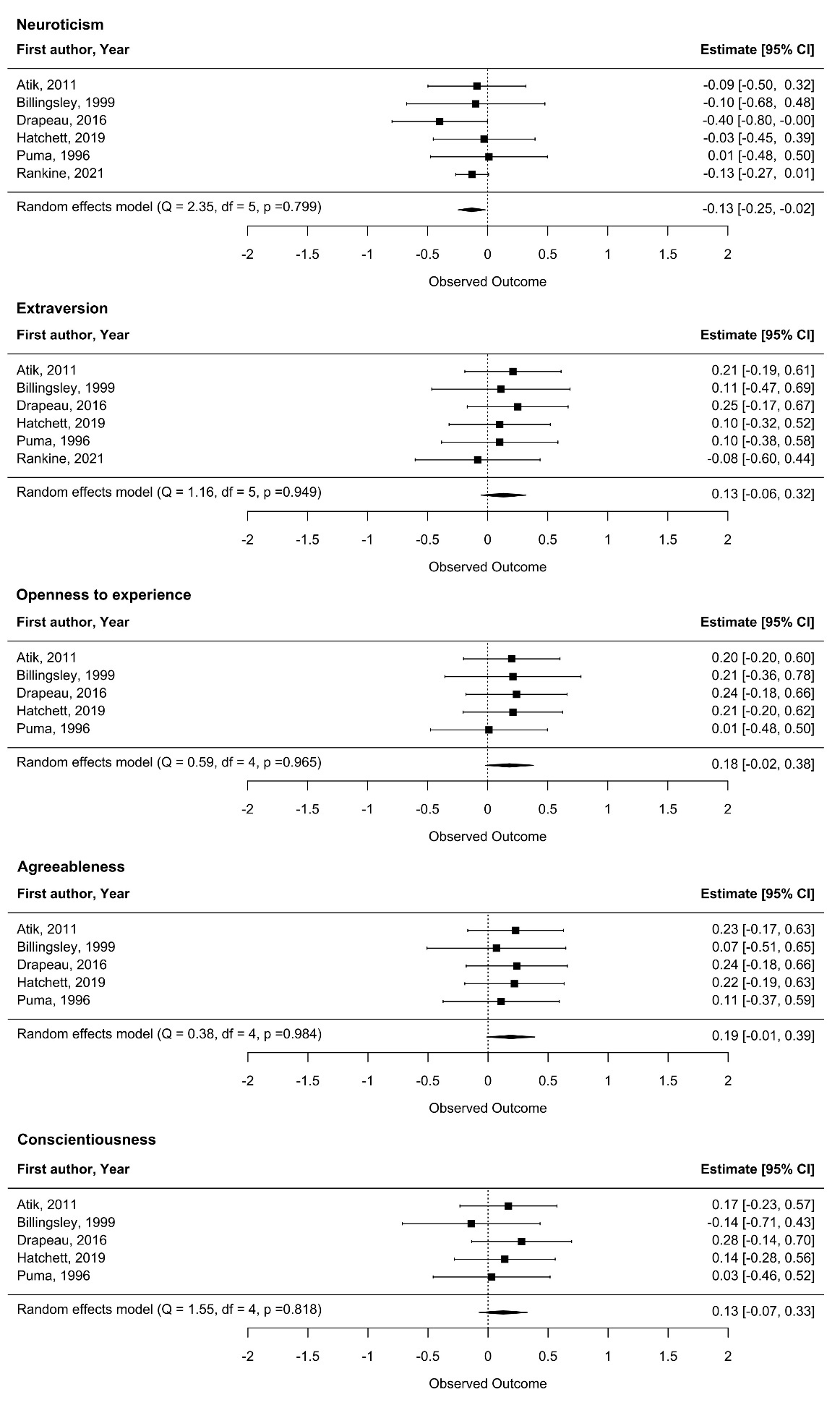

Supplement: Supplementary file 1 [file DataSheet1.docx]
